# Antifungal and Anti-Oomycete Potential of Ag_2_S-S Janus and Ag Nanoparticles Synthesized with Non-Toxic Agents and Their Application to Control *Fusarium* Wilt of Tomato

**DOI:** 10.3390/ijms27177916

**Published:** 2026-09-04

**Authors:** Rosa Elvira Sánchez-Fernández, Moisés Camacho Tapia, Daniel Canseco-González, Guadalupe Stefanny Aguilar-Moreno, Miguel Angel Aguilar-Méndez, Francisco Ascencio, Leticia Rojas-Sandoval, Elizabeth Navarro Cerón

**Affiliations:** 1 Laboratorio Nacional de Investigación y Servicio Agroalimentario y Forestal (LANISAF), Universidad Autónoma Chapingo, Carretera México-Texcoco km 38.5, Chapingo, Texcoco 56230, Estado de México, Mexico; resf2012@gmail.com; 2 Departamento de Parasitología, Universidad Autónoma Chapingo, Carretera México-Texcoco km 38.5, Chapingo, Texcoco 56230, Estado de México, Mexico; moises.camachotapia@gmail.com; 3 SECIHTI-Laboratorio Nacional de Investigación y Servicio Agroalimentario y Forestal, Universidad Autónoma Chapingo, Texcoco 56230, Estado de México, Mexico; jdcanseco@secihti.mx; 4 Secretaría de Ciencia, Humanidades, Tecnología e Innovación (SECIHTI)—Universidad Autónoma Chapingo, Carretera México-Texcoco km 38.5, Chapingo, Texcoco 56230, Estado de México, Mexico; stefanny.aguilar.m@gmail.com; 5 Instituto Politécnico Nacional, Centro de Investigación en Ciencia Aplicada y Tecnologıía Avanzada, Legaria 694, Colonia Irrigación, Mexico City 11500, Mexico; maguilarme@ipn.mx; 6 Departamento de Fitotecnia, Universidad Autónoma Chapingo, Carretera México-Texcoco km 38.5, Chapingo, Texcoco 56230, Estado de México, Mexico; letizia1234rojaz@gmail.com

**Keywords:** silver nanoparticles, Janus nanoparticles, plant pathogenic fungi, antifungal activity, *Fusarium* wilt, tomato plants

## Abstract

The excessive use of fungicides in agriculture has generated environmental problems, increased resistance in plant pathogenic fungi, and raised concerns for food safety and human health. This study evaluates silver-based nanoparticles (NPs) as a potential alternative for the control of plant pathogens. Ag_2_S-S Janus and AgNPs were synthesized through chemical reduction using non-toxic compounds, employing glucose as a reducing agent (0.30 and 0.90%) and gelatin as a passivating agent (0.50 and 1.00%), generating four treatments. The synthesized NPs showed average sizes ranging from 5 to 11 nm, with the smallest particles obtained in treatment T3 (5.90 ± 0.30 nm) and the largest in T4 (10.34 ± 0.46 nm). The antifungal and anti-oomycete activity of the NPs was evaluated in vitro against four microorganisms: *Alternaria alternata*, *Colletotrichum boninense*, *Fusarium oxysporum*, and *Phytophthora capsici*. Results showed complete inhibition (100%) of *A. alternata* and 60–70% inhibition of *P. capsici* in all treatments, demonstrating strong antimicrobial potential. Additionally, NPs were applied to tomato seedlings (*Solanum lycopersicum*) inoculated with *Fusarium oxysporum* f. sp. *lycopersici*. NPs were not phytotoxic and reduced disease severity (on a 1–2 scale) while promoting lesion healing.

## 1. Introduction

Global agricultural production has intensified in response to the increasing demand for food, feed, and raw materials. However, crop productivity remains severely constrained by pests and diseases, among which plant-pathogenic fungi are particularly destructive, causing substantial yield and quality losses. In addition, certain fungal species produce mycotoxins that pose significant risks to human health [1]. In conventional crop management, excessive fungicide applications lead to resistance in phytopathogenic fungi and to environmental contamination [2,3]. Therefore, there is an urgent need to develop sustainable and environmentally friendly alternatives that minimize the risk of resistance development.

Nanotechnology has emerged as a promising approach for plant disease management. Various nanomaterials (such as Ag, Ag_2_S, Cu, CuO, ZnO, TiO_2_, and Janus nanoparticles [NPs]) have demonstrated antimicrobial properties [4,5,6,7,8,9,10]. Among these, silver nanoparticles (AgNPs) have attracted particular attention due to their strong inhibitory activity against fungi, bacteria, and viruses [9]. AgNPs have been reported to suppress the growth of several phytopathogenic fungi, including *Rhizoctonia solani*, *Fusarium oxysporum*, *Fusarium verticillioides*, *Sclerotinia sclerotiorum*, *Sclerotium rolfsii*, *Alternaria brassicicola*, *Colletotrichum gloesporioides*, *Bipolaris oryzae*, *Uromyces viciafabia*, *Ustilago hordei*, and *Ustilago maydis* [10,11,12]. Furthermore, asymmetric Ag/Cu Janus nanoparticles have shown enhanced antifungal activity, including effective inhibition of *R. solani* [6].

Previous studies have demonstrated that the antifungal efficacy of AgNPs is due to their Ag^+^ ion release and their catalytic oxidation abilities. It also depends on physicochemical properties such as size, morphology, and concentration [7,13]. For instance, Akpinar et al. [7] observed inhibition of mycelial growth of *F. oxysporum* f. sp. *radicis-lycopersici* in vitro by applying different concentrations of AgNPs to agar plates previously cultured with the fungus, with the smallest NPs (3 nm) being the most active; likewise, the antifungal activity is dependent on the concentration of AgNPs. Notably, *Fusarium oxysporum* f. sp. *lycopersici* (Fol), the causal agent of *Fusarium* wilt in tomato, is one of the most economically important pathogens, leading to yield losses of 21–47% in open-field and protected cultivation systems [14,15].

Ag_2_S NPs also exhibit antifungal activity against *F. verticillioides*, *B. oryzae*, *U. hordei,* and *U. viciafabia* and, therefore, their combination with AgNPs could potentially result in a synergistic antifungal effect. Moreover, sulfur is an essential macronutrient with well-documented antifungal properties, which may further contribute to the antifungal activity of Ag_2_S-based nanomaterials [10,13].

Beyond their antimicrobial activity, AgNPs have also been associated with beneficial effects on plant physiology. These include enhanced seedling growth, induction of resistance-related gene expression [16], and modulation of key physiological processes such as water and nutrient uptake, photosynthesis, respiration, and transpiration. Additionally, AgNPs can influence oxidative stress responses by regulating the production of reactive oxygen species (ROS) and by activating antioxidant defense systems [17,18,19,20].

Although concerns have been raised regarding the potential phytotoxicity and environmental impact of nanoparticles, these effects can be mitigated through careful control of their size, shape, and surface properties. Additionally, Ag_2_S is considered one of the least toxic forms of silver due to its slow release of Ag^+^ ions into the environment [10,21,22,23,24].

In this context, the present study reports the synthesis of four Ag_2_S-S Janus and AgNP formulations (T1–T4) by chemical reduction, their physicochemical characterization, and their antifungal and anti-oomycete effects against the fungi *Alternaria alternata*, *Colletotrichum boninense*, *F. oxysporum*, and the oomycete *Phytophthora capsici*. Additionally, the protective effect of NPs was assessed in tomato seedlings inoculated with Fol.

## 2. Results and Discussion

### 2.1. Nanoparticle Synthesis

Ag/Ag_2_S nanoparticles were synthesized. First, Ag^+^ ions in aqueous solution react with hydroxyl ions to form silver oxide (Ag_2_O). Next, AgNPs synthesis is attributed to the reduction in Ag_2_O by glucose under alkaline conditions (pH = 10). The dark brown color after adding AgNO_3_ is consistent with the formation of metallic AgNPs. Gelatin stabilizes the nanoparticles by interacting with the Ag^0^ surface through its functional groups, thereby limiting particle aggregation [25]. Additionally, sulfur-containing amino acid residues in gelatin serve as a source of sulfur species that can react with Ag_2_O, promoting the formation of Ag_2_S NPs (see Equations (1) and (2)).

### 2.2. UV-Vis Spectroscopy

**Figure 1:**
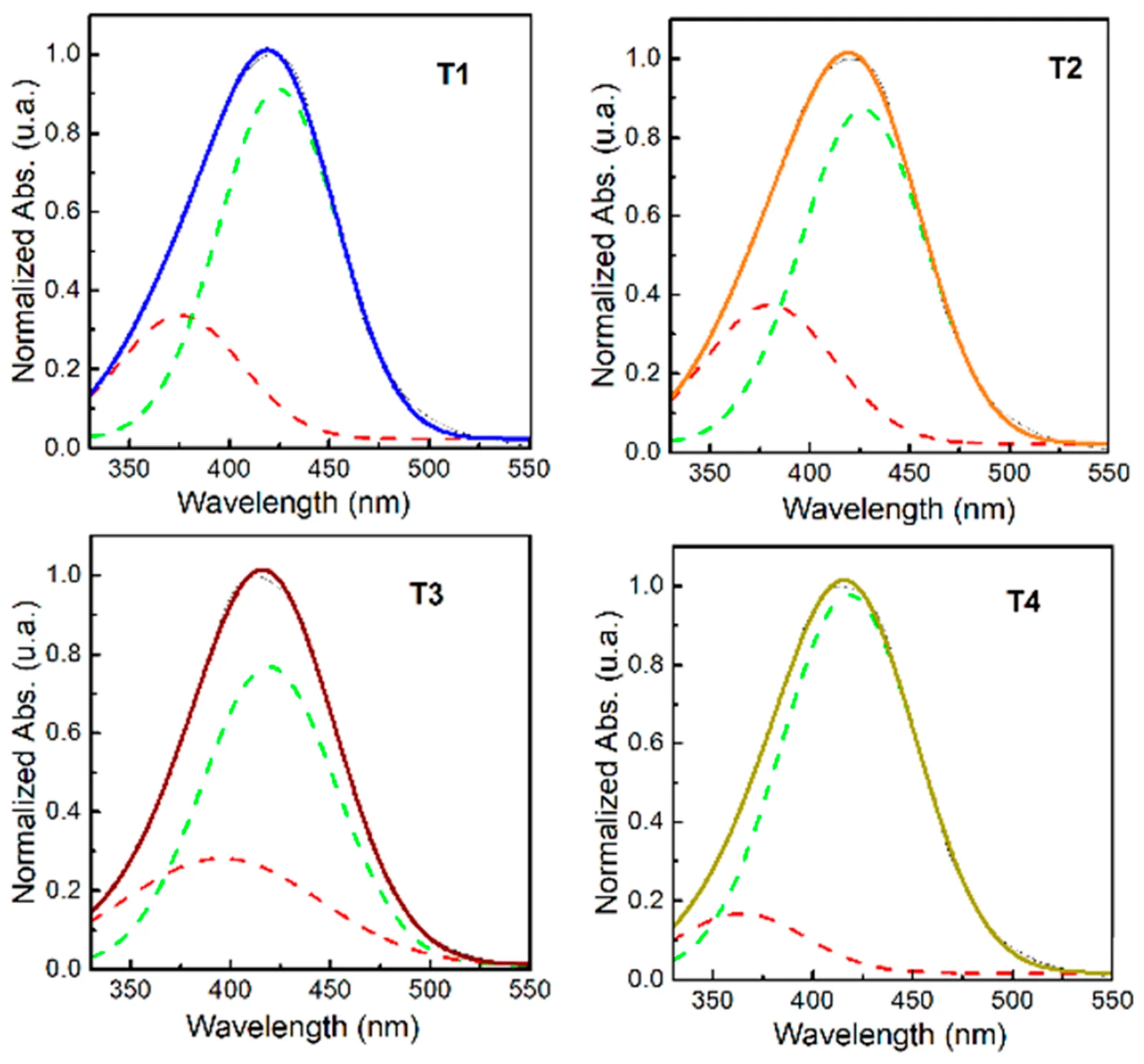
Absorbance spectra of the samples T1–T4. The red dotted lines correspond to the contribution of Ag_2_S, and the green dotted line corresponds to the plasmonic contribution due to Ag.

Figure 1 shows the UV-Vis spectra of the four NPs treatments. The bands exhibit a very pronounced asymmetry related to at least two contributions. After the deconvolution of the contribution, we observed the most intense band related to the Ag plasmonic peak between 418.16 and 426.67 nm. These values are characteristic of the surface plasmonic resonance of AgNPs [26]. Therefore, it is confirmed that glucose, as an aldehyde, could reduce the Ag^+^ ions to silver atoms and, subsequently, the formation of the NPs. A small shift in the wavelength of the surface plasmon resonance band is related to obtaining silver NPs of different shape and size [27]. If the particle size increases, the absorption band tends to have longer wavelengths. Likewise, the maximum absorption peak broadens, as the silver NPs are not symmetrical [25]. The second band is related to the Ag_2_S acanthite phase between 364.33–395.51 nm. This absorbance reported by Sibiya and Moloto (2018) [28] is consistent with the absorption band observed in our study.

### 2.3. X-Ray Diffraction (XRD)

**Figure 2:**
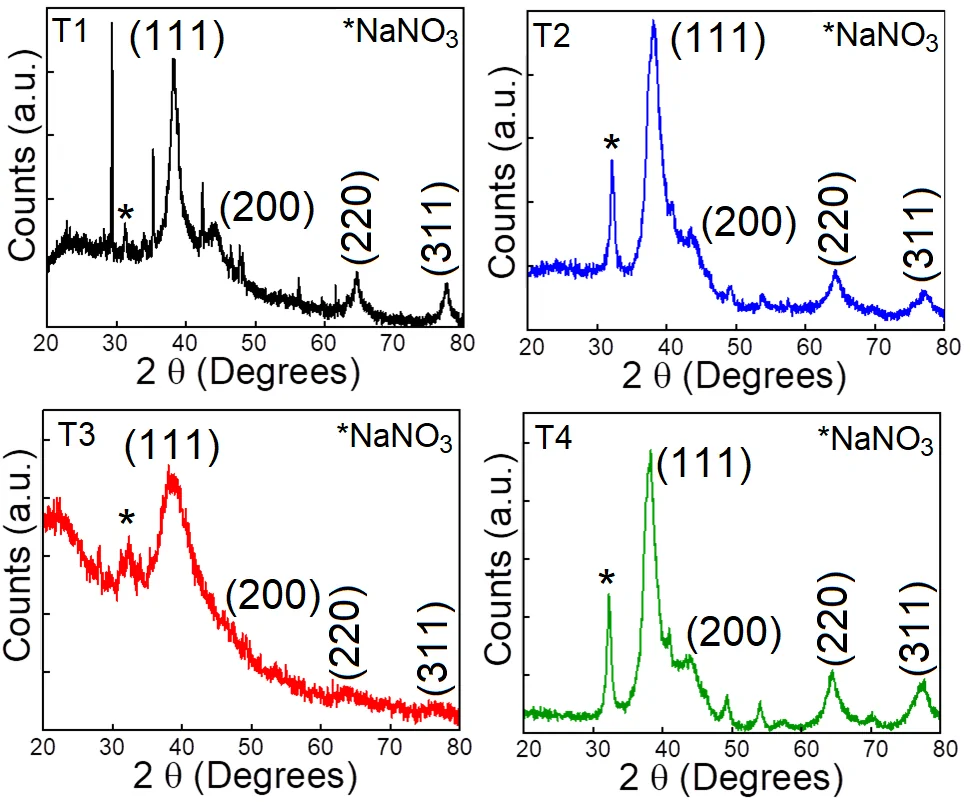
X-ray diffraction (XRD) patterns for different samples from T1 to T4 of Ag/Ag_2_S nanoparticles.

The crystalline structure of the powder samples produced (T1–T4) at different temperatures after the gelatin synthesis process was analysed by XRD. Figure 2 shows the XRD patterns of the four samples. Clearly defined diffraction peaks were observed at 2θ values of ~38.01°, 44.21°, 64.39° and 74.40°, which can be readily indexed as the (111), (200), (202) and (311) crystal planes related to the Ag-FFC structure in the Fm3m (225) space group. The Crystallography Open Database (COD) card number is 96-110-0137. Additionally, multiple peaks related to secondary phases associated with Ag_2_S (acanthite COD = 96-150-9711 and argentite COD = 96-101-1338), as well as AgCl COD = 96-901-1680 and NaNO_3_ COD = 96-810-3616 residues, were observed. The crystal size was determined from the diffractograms using the Debye-Scherrer formula [29]. Table 1, which summarises the XRD results, is shown below.

The Ag_2_S Janus NPs, acanthite and argentite, are present at 37.9% and 31.1%, respectively, with Ag at 6.5% and AgCl at 24.5% in treatments T1, T2, and T4. In the case of treatment T3, which exhibited the highest biological activity, the composition is as follows: Ag_2_S acanthite (34.1%) and argentite (38.1%), Ag 5.8%, and AgCl 22.0%.

Simultaneous formation of metallic Ag and Ag_2_S NPs may account for the coexistence of these phases in the synthesized material. The AgCl phase detected by XRD may result from the reaction of residual Ag^+^ with trace chloride ions. Therefore, the final samples are a multiphase of the Ag_2_S NPs as the main reduction product, together with a Ag system containing AgNPs and AgCl as secondary phases and a NaNO_3_ phase as a by-product of the reaction.

### 2.4. Morphological Characterization and Chemical Composition

**Figure 3:**
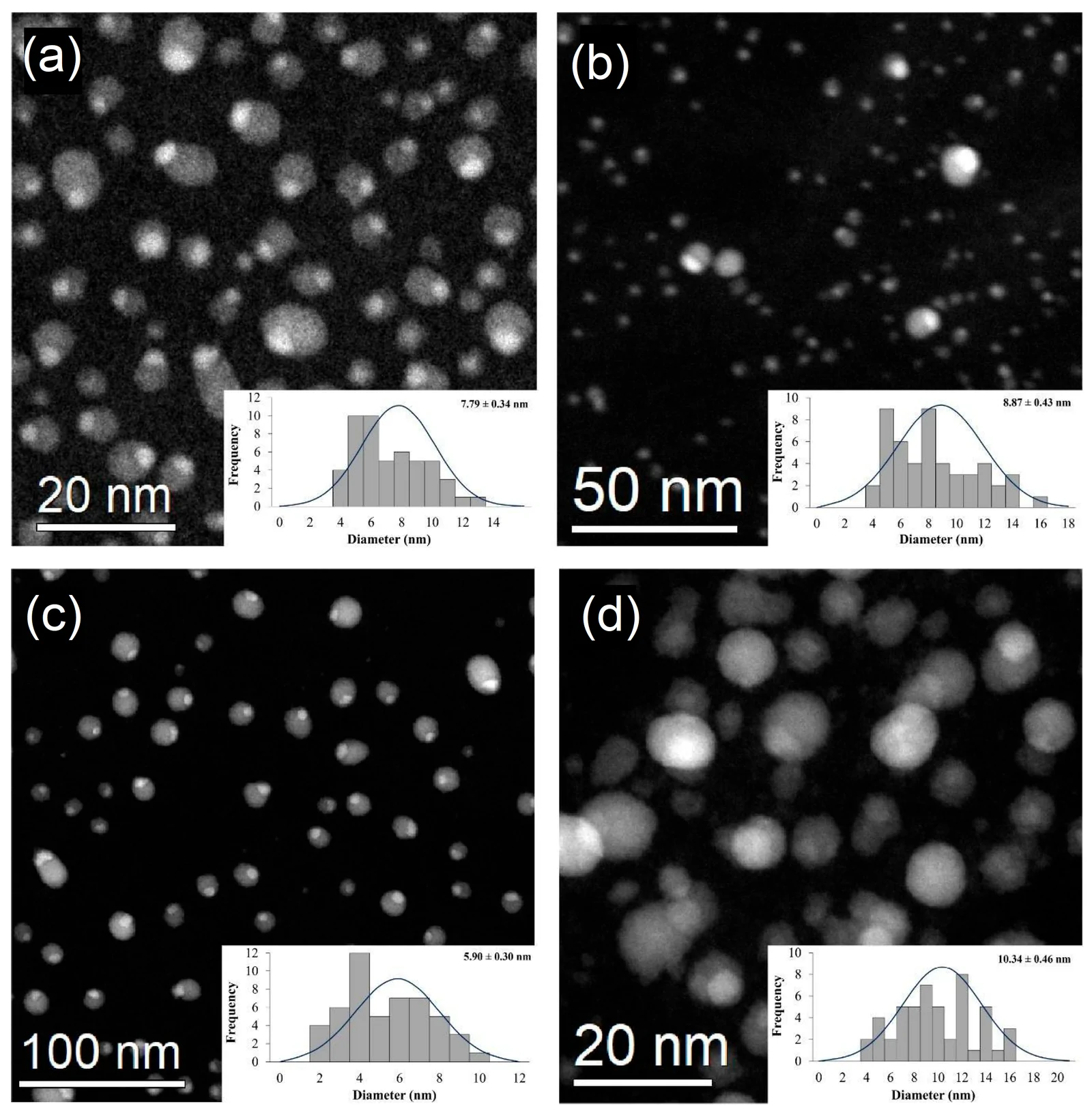
HAADF micrographs and histograms of size distribution of 50 nanoparticles synthesized under the different conditions: (**a**) 0.30% glucose and 0.50% gelatin (T1), (**b**) 0.30% glucose and 1.00% gelatin (T2), (**c**) 0.90% glucose and 0.50% gelatin (T3), and (**d**) 0.90% glucose and 1.00% gelatin (T4).

The NPs obtained presented homogeneous Janus morphology and a relatively narrow size distribution (Figure 3a). The size distribution of NPs was determined by measuring the diameter of 50 particles on the STEM images. The synthesis conditions of T3 treatment favored the production of NPs with a mean size of 5.90 ± 0.30 nm, where most of the NPs are in the 4 to 5 nm range (Figure 3c). On the contrary, the largest NPs size was obtained with T4, presenting a mean diameter of 10.34 ± 0.46 nm and a slightly heterogeneous distribution, with NPs from 4 to 16 nm (Figure 3d). The difference between T3 and T4 is the gelatin amount (0.5 and 1.0%, respectively), which indicates that a lower concentration of gelatin works better as a passivating agent in the synthesis of Ag_2_S-S NPs.

The values obtained are lower than those reported by Aguilar-Méndez et al. [25], who obtained particles of 5.8 ± 2.4 nm and 24 ± 6.9 nm, with the same type of synthesis and precursors. Alzahrani et al. [30] obtained AgNPs ranging from 11.9 to 38.8 nm by green synthesis, which is much higher than that reported in this work. Although the synthesis utilized in the present work is not green, it can be considered an environmentally friendly technique due to the precursors and the synthesis method used.

**Figure 4:**
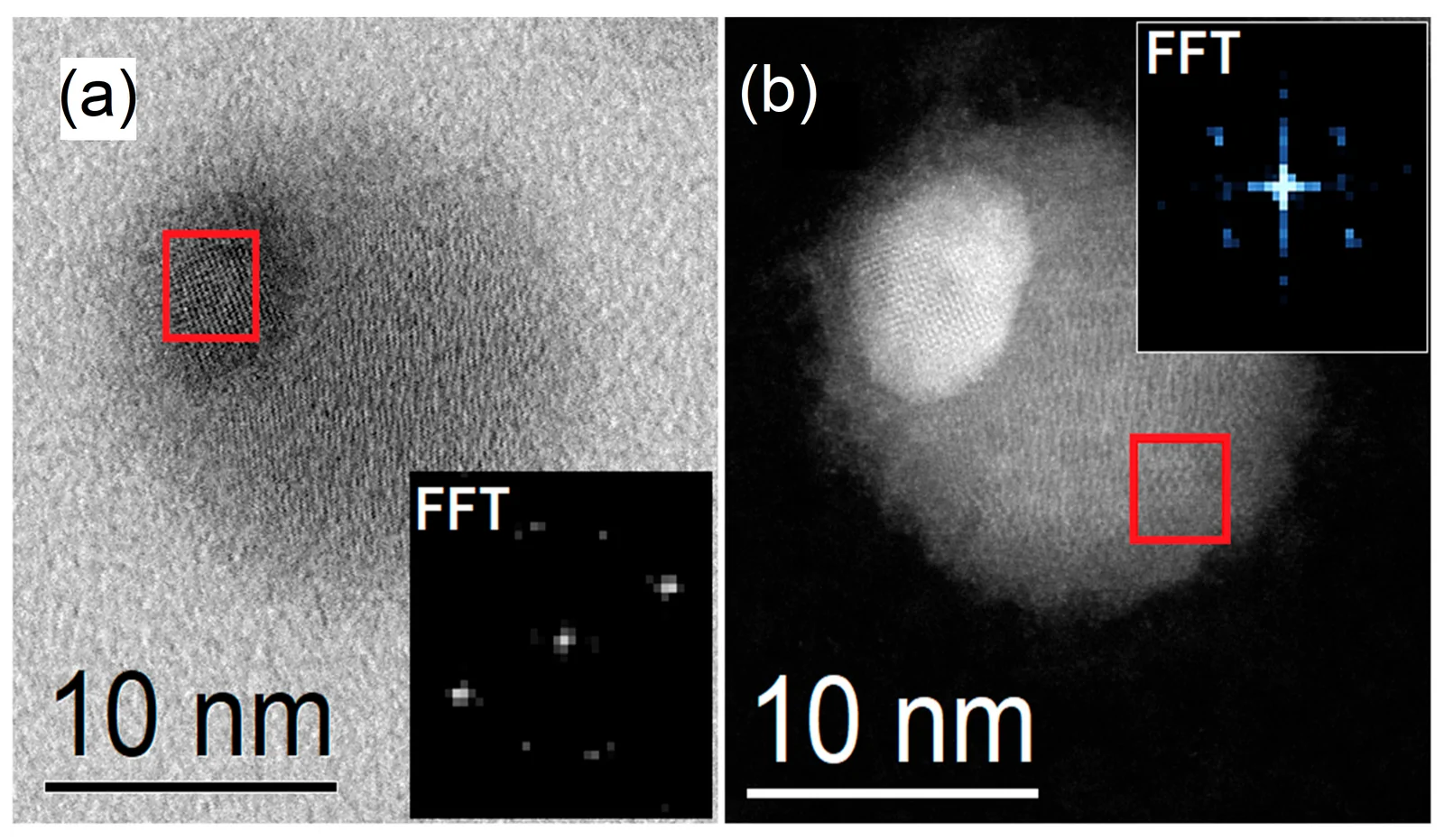
(**a**) HRTEM image of an Ag_2_S nanoparticle (sample T2) with the corresponding FFT, and (**b**) high-resolution HAADF image of a single nanoparticle with the FFT. (**b**) shows a sulfur phase.

A detailed study of the morphology and size of the nanoparticles was performed by HRTEM. Figure 4a shows the HRTEM image of a representative isolated Janus nanoparticle of acanthite Ag_2_S with a size of 15 nm, the inset shows the fast Fourier transform (FFT), and from the FFT analysis, space group P121/c1 (14) was determined. The measured interplanar distances were 0.254, 0.252, and 0.191 nm, corresponding to the crystallographic planes ($1\bar{11}$), (101), and (011) using the crystallographic reference COD: 96-150-9711, oriented along the zone axis [121]. Figure 4b shows a high-resolution HAADF (High-Angle Annular Dark-field), in which the analysis shows that the FFT of the red box corresponds to the sulfur phase.

**Figure 5:**
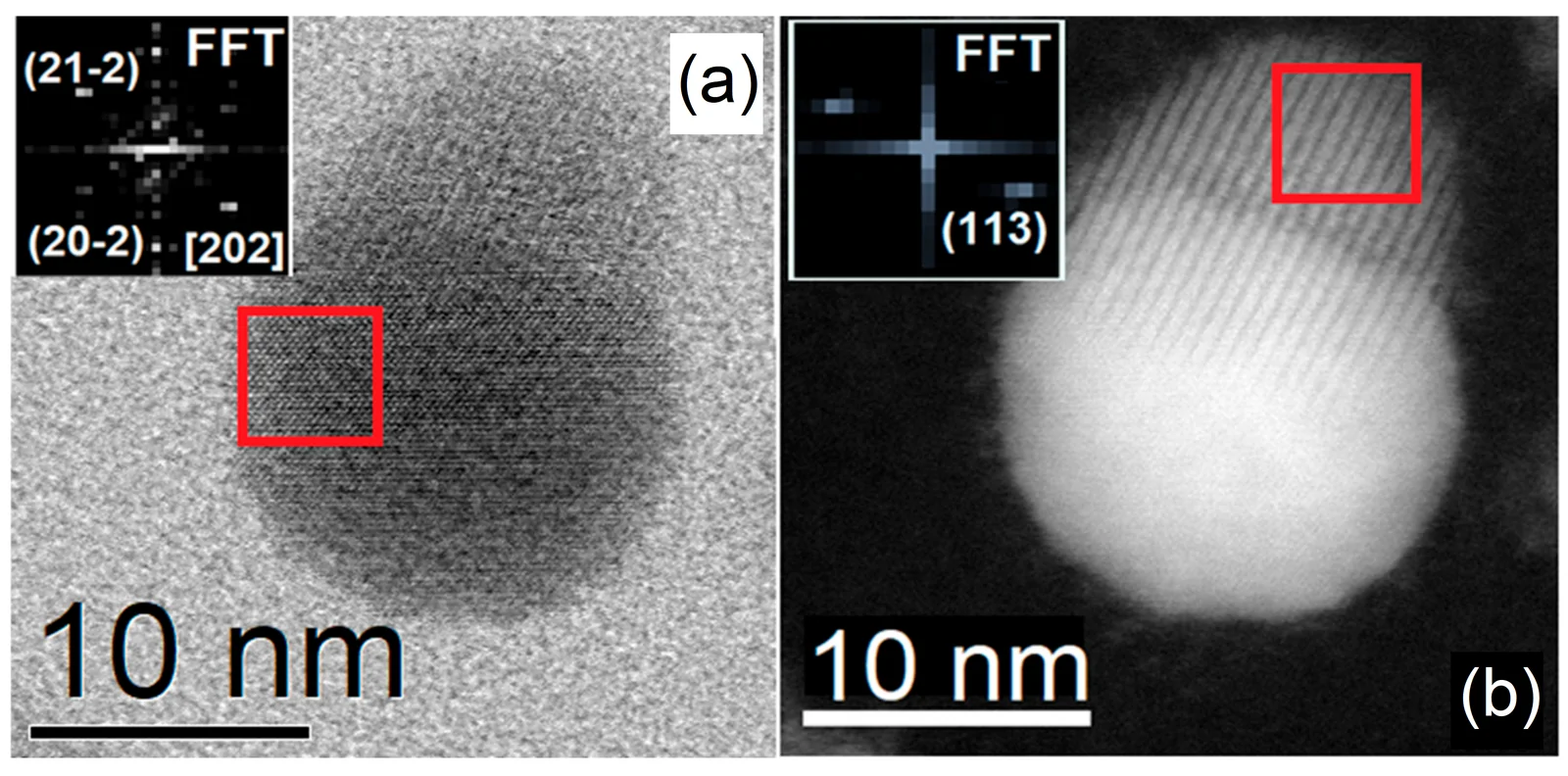
(**a**) HRTEM image of an Ag_2_S-S nanoparticle (sample T3) with the corresponding FFT, and (**b**) high-resolution HAADF image of a single nanoparticle with the FFT.

Figure 5a shows another particle sample with an average size of 14 nm, and the inset shows the FFT of the outlined red area. This particle also has the Ag_2_S crystal structure, and each diffraction corresponds to the (2,0,−2) and (2,1,−2) crystallographic planes using the same crystallographic reference. The interplanar distances were 0.287, 0.252, and 0.193 nm, respectively. This particle is oriented along the zone axis [1,0,1].

**Figure 6:**
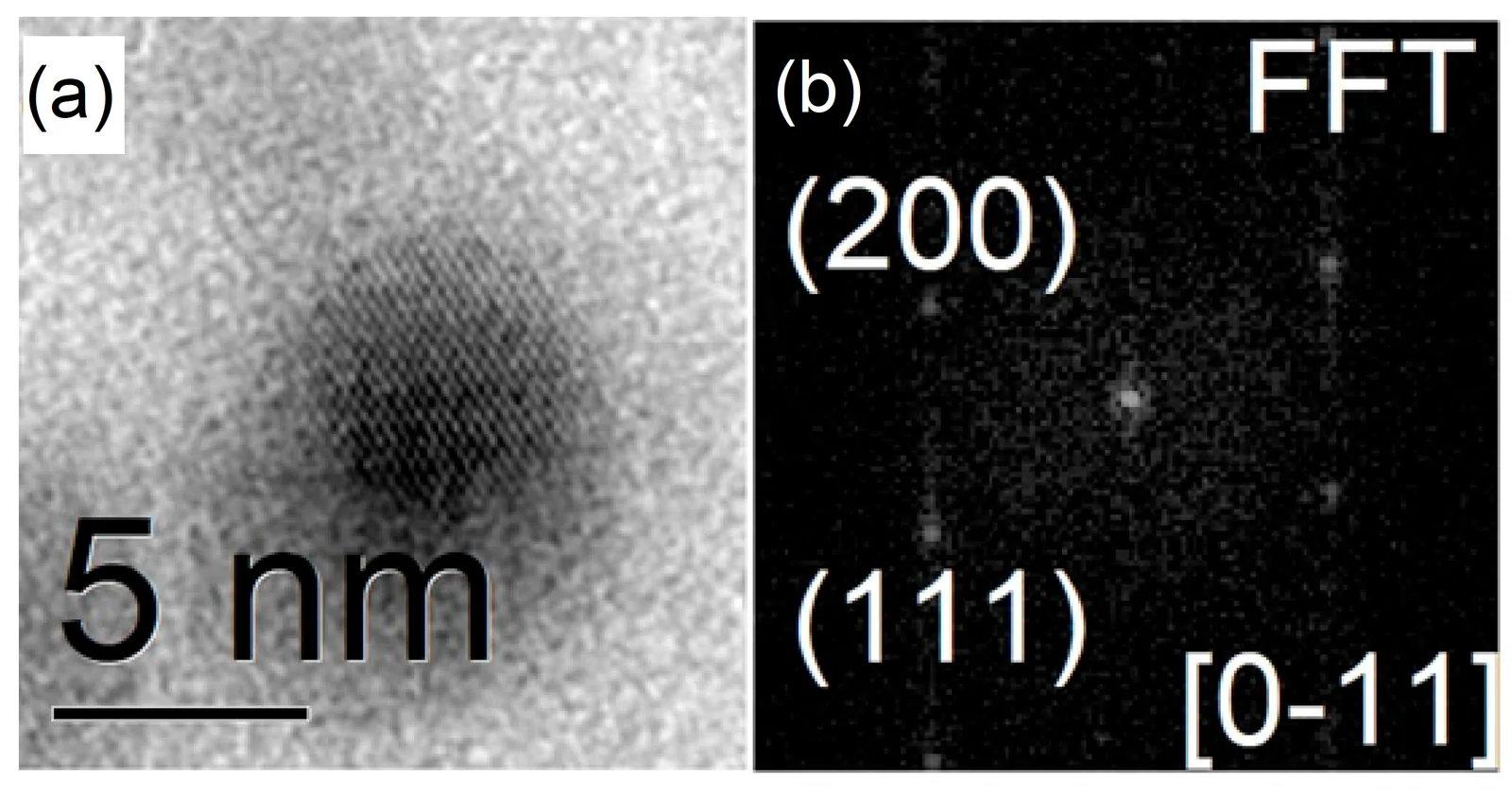
HRTEM image of an individual small Ag nanoparticle (**a**) and its corresponding FFT (**b**).

In addition to the Janus Ag_2_S-S nanoparticles, some Ag particles were detected in smaller quantities. Figure 6 shows the HRTEM image of an isolated Ag particle in which it is observed a single crystal structure of a size of 5 nm. The inset of the figure, the FFT shows the crystallographic planes corresponding to the (2,0,0) and (1,1,1) of the FCC Ag- COD 96-110-0137, with d_200_ = 0.239 nm and d_111_ = 0.269 nm, respectively. This particle is oriented in the [0,−1,1] direction.

**Figure 7:**
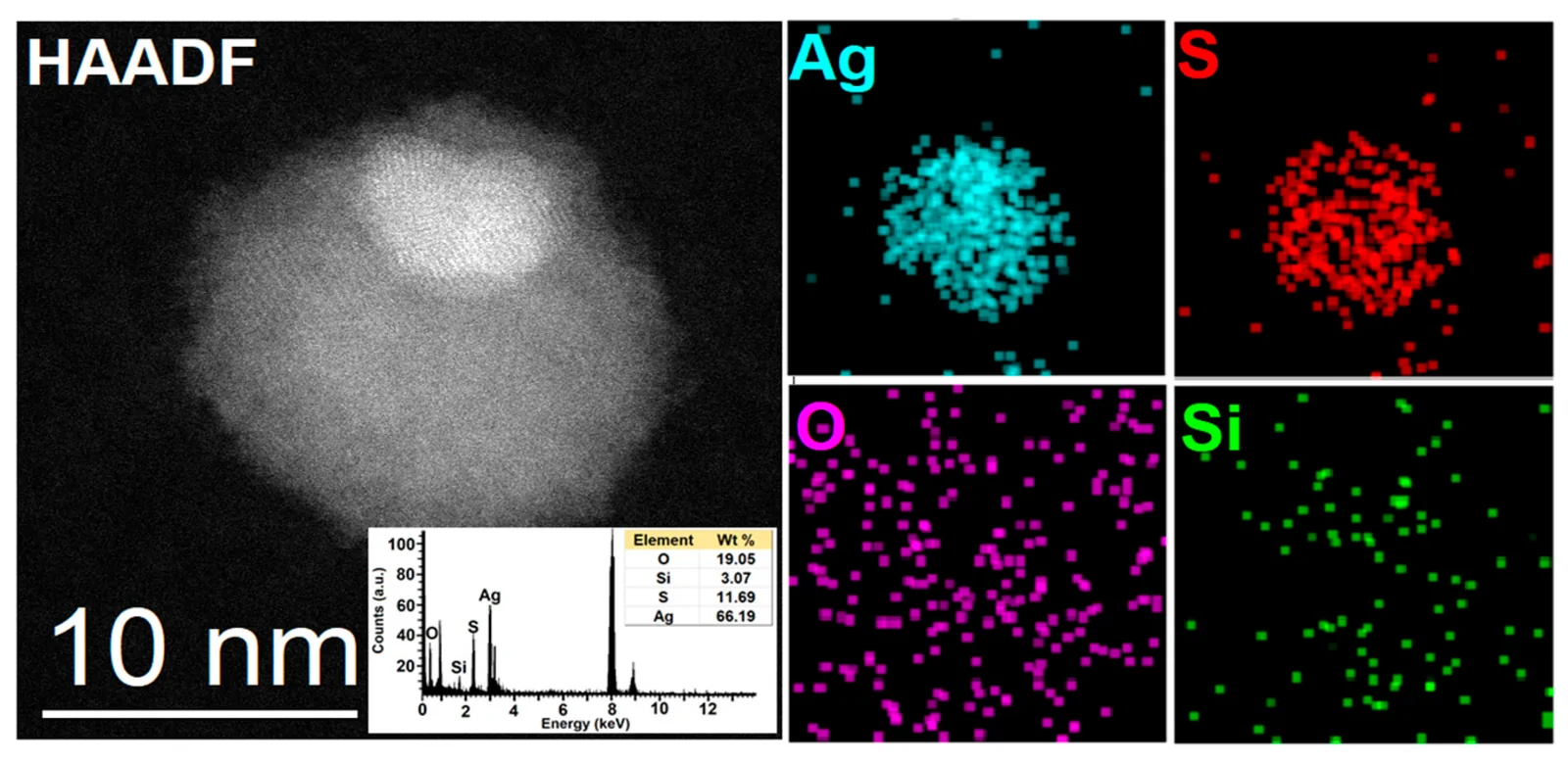
HAADF-STEM and element mapping images of the Ag_2_S-S Janus nanoparticle.

In Figure 7, chemical mapping was carried out to obtain detailed information about the composition of the nanoparticles, and HAADF-STEM and energy-dispersive spectroscopy (EDS) analyses were carried out in the inset. One Ag_2_S-S Janus NPs is observed, in which two phases are clearly distinguished, the first related to Ag_2_S and the second to S. It is observed that a more significant amount of Ag is segregated in the upper part. EDS mapping confirms the Ag_2_S-S Janus structure and a segregated Ag phase. The presence of Ag, S, O, and Si is confirmed in the Ag_2_S phase. The weight percentage of the elements was: 66.19% Ag, 11.69% S, 19.05% O, and 3.07% Si. Their spatial distribution is shown in the elemental maps. The large amount of oxygen detected is attributed to products of the aqueous synthesis process, and the Cu signal detected is related to the copper grid.

### 2.5. Dynamic Light Scattering (DLS) and Z-Potential Analysis

Dynamic light scattering (DLS) was used to determine the hydrodynamic diameter and polydispersity index (PDI) of the NPs synthesized in an aqueous colloidal medium (Table 2). According to the results, the treatments with the lowest gelatin concentration (0.5%) exhibited the smallest hydrodynamic diameters (131 ± 24 and 137 ± 0.5 nm), whereas increasing the gelatin concentration to 1.0% promoted particle growth, resulting in values of 402 ± 16 and 602 ± 51 nm. This behavior can be attributed to gelatin’s role as a stabilizing agent and surface coating for the nanoparticles. At higher concentrations, a greater number of gelatin molecules can adsorb onto the surface of the nanoparticles, forming a thicker organic layer that increases the hydrodynamic diameter determined by DLS [31,32].

Furthermore, high concentrations of this biopolymer could promote particle–particle interactions or aggregation phenomena mediated by the gelatin chains, contributing to an increase in the apparent size of the nanoparticles in suspension [33].

The hydrodynamic diameter obtained by DLS is inherently larger than the size of the metal core observed by TEM. This discrepancy was expected due to the different measurement principles involved. While DLS measures the apparent size of solvated nanoparticles undergoing Brownian motion—including the inorganic core, the organic coating layer, and the associated hydration layer—the TEM technique allows only the size of the dehydrated, crystalline silver core to be determined [34].

Meanwhile, the PDI values for treatments 1 and 3 (0.22 and 0.19, respectively) indicate a relatively narrow size distribution, while treatments 2 and 4 had values of 0.38 and 0.51, respectively, indicating greater heterogeneity in the particle population. This behavior is consistent with the larger hydrodynamic diameters observed in those treatments and suggests that the increase in gelatin concentration favored the formation of colloidal systems with a broader size distribution, possibly as a result of differences in the thickness of the polymeric coating or of partial aggregation phenomena. According to the literature, PDI values greater than 0.7 reflect highly polydisperse systems, while values less than 0.3 typically indicate moderate to good monodispersity [35].

The surface charge of the synthesized colloidal NPs was evaluated using zeta potential measurements (Table 2), a critical parameter for determining the stability of nanoparticles in suspension. According to Seema et al. [36], dispersions with zeta potential values between ±0 and ±10 mV are considered highly unstable; between ±10 and ±20 mV, relatively unstable; between ±20 and ±30 mV, moderately stable; and values greater than ±30 mV, highly stable.

Based on these criteria, among the four treatments obtained in this study, only T3 exhibited moderate colloidal stability, with a zeta potential of −23 ± 2.8 mV. This result is consistent with its low PDI value (0.19), suggesting a more homogeneous size distribution and greater dispersion stability compared to the other treatments.

### 2.6. Antifungal and Anti-Oomycete Activity of Ag_2_S-S and Ag Nanoparticles

**Figure 8:**
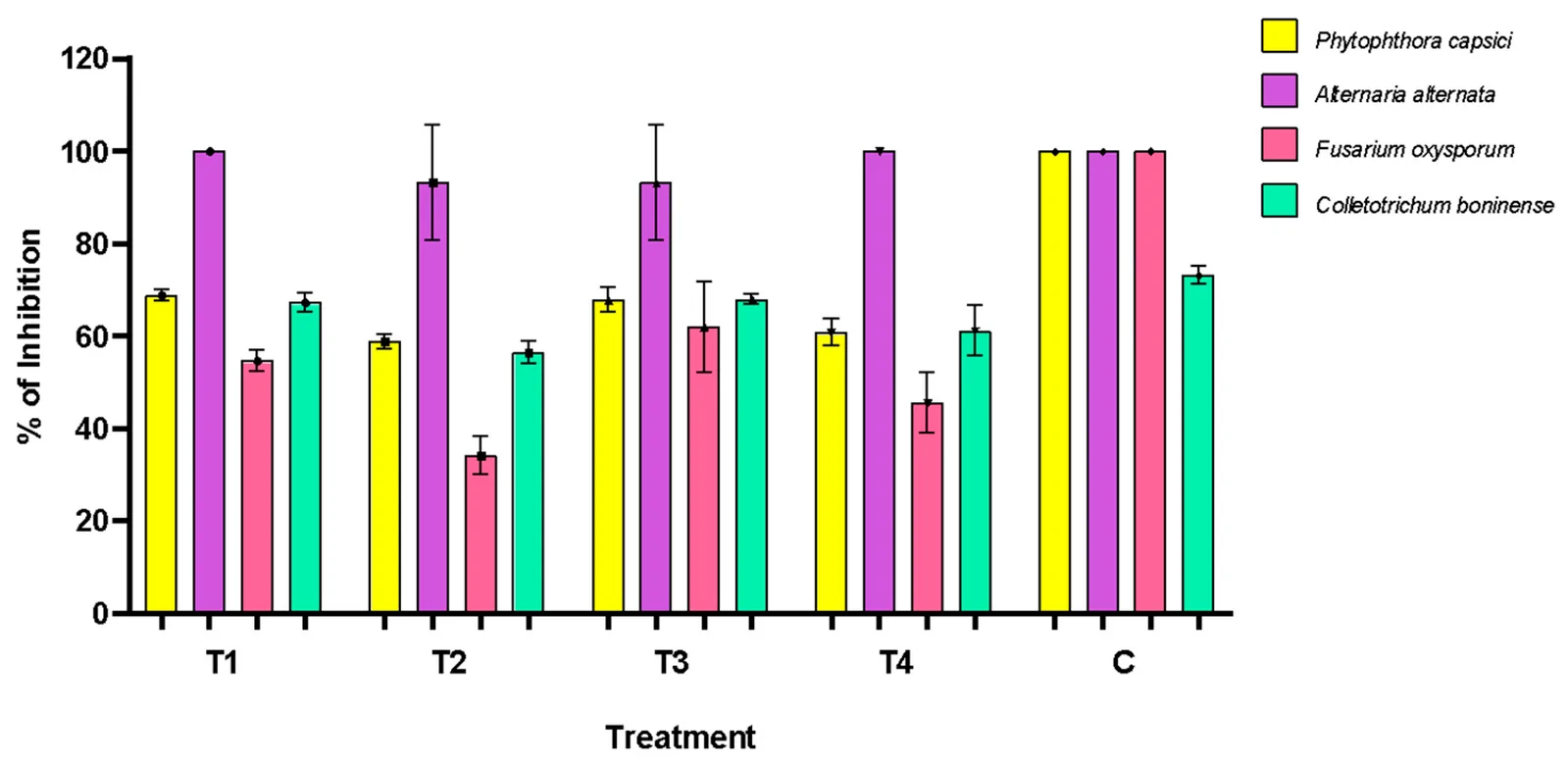
Inhibition percentages after seven days of evaluation of the antifungal and anti-oomycete activity of four Ag_2_S-S Janus and AgNPs (T1–T4) and the chemical control (C) at a concentration of 100 µg∙mL^−1^ on the growth of four phytopathogenic microorganisms. Chemical control: Captán 50^®^ for fungi and Ridomil Gold^®^ 480 SL for oomycetes. Data are represented as mean ± standard deviation (SD). All treatments were statistically significant (Tukey, *p* < 0.05).

**Figure 9:**
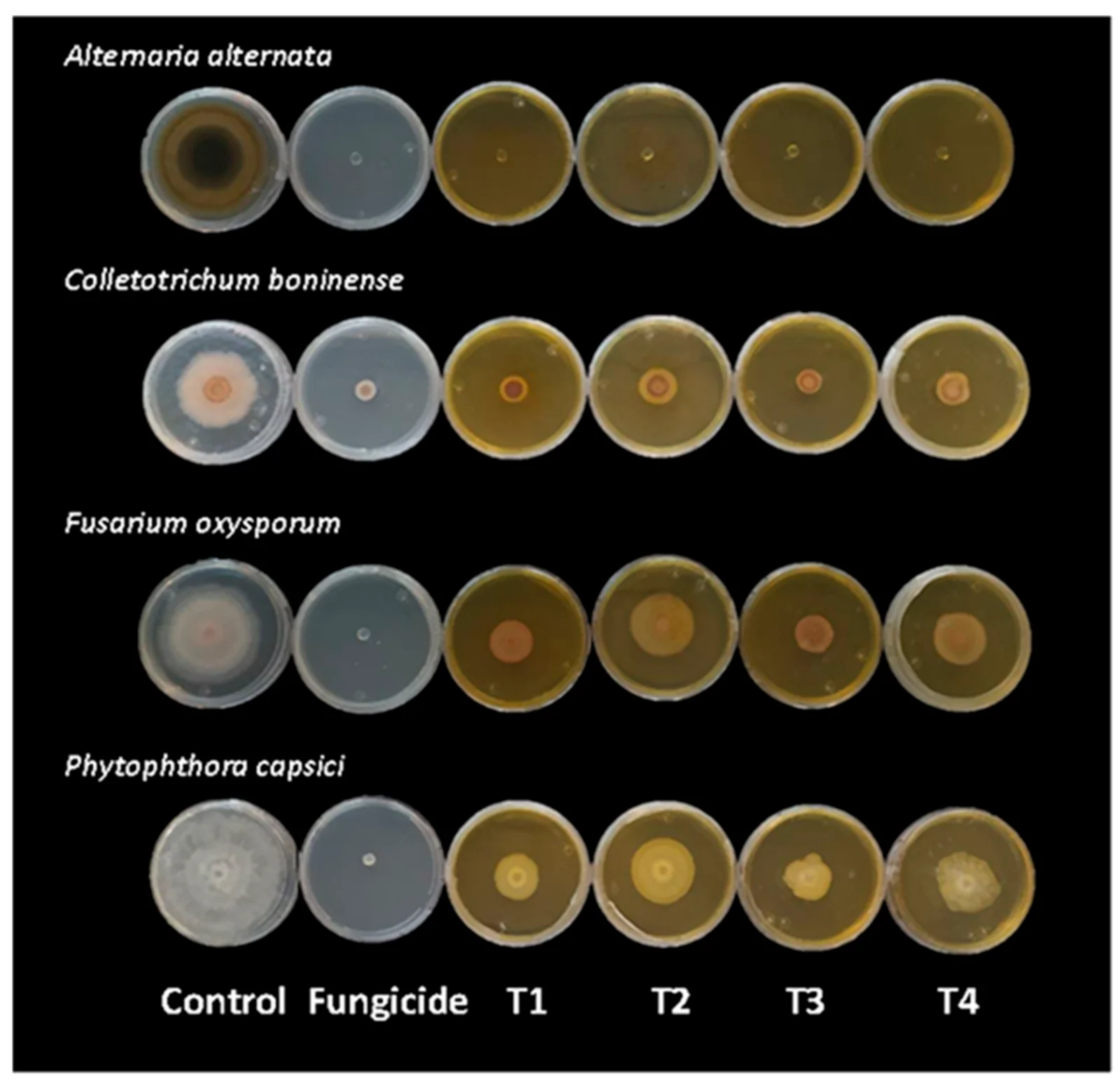
The antifungal and anti-oomycete activity observed 7 days after the evaluation of four Ag_2_S-S Janus and AgNPs (T1–T4) at a concentration of 100 µg∙mL^−1^ against three fungi and one oomycete, using as a control Captán 50^®^ fungicide, which was positive for fungi, and Ridomil Gold^®^ 480 SL for oomycetes, and PDA culture medium was used as a negative control.

In the antifungal and anti-oomycete activity assay of four NPs with different physicochemical properties against the plant pathogenic microorganisms *A. alternata*, *C. boninense*, *F. oxysporum*, and *P. capsici*, a significant inhibition of the microorganisms’ growth was observed, with inhibition percentages ranging from 30 to 100% at seven days of evaluation. In the case of *A. alternata*, all treatments completely inhibited the growth of the fungus (Figure 8). Likewise, it can be observed that the morphology of the *Fusarium* and *Phytophthora* colonies changes with the NPs treatment (Figure 9).

The smallest NPs, T1 (7.79 nm) and T3 (5.9 nm), showed a better effect on mycelial growth. Smaller AgNPs have a larger surface area and better biological activity [7,37]. The shape of NPs can also significantly influence their antifungal activity. One study directly compared spherical, cubic, and wire-shaped AgNPs against the fungi *Candida albicans*, *Candida glabrata*, and *Candida tropicalis*. Among the three morphologies, cubic AgNPs exhibited the highest antifungal activity, followed by spherical and wire-shaped AgNPs. However, this difference may also be partially attributed to particle size, as the nanowires ranged from 250 to 500 nm, whereas the other AgNPs were approximately 30–50 nm in size [13,38].

In the present study, our formulations consist of a mixture of Ag_2_S–S Janus NPs and AgNPs. Janus nanoparticles are anisotropic nanostructures composed of two distinct compartments or faces that differ in polarity and/or chemical composition [39]. In contrast, multiply twinned silver nanoparticles (MT-AgNPs) represent one of the most extensively studied classes of structurally engineered silver nanomaterials, owing to the distinctive optical and electronic properties of silver. These particles commonly adopt decahedral (10-faced) or icosahedral (20-faced) geometries, stabilized by a network of shared {111} crystallographic twin planes. In addition, the composition of the colloidal mixture is also important: Ag_2_S Janus NPs in the form of acanthite and argentite are present at 37.9% and 31.1%, respectively, with AgNPs at 6.5% and AgCl at 24.5% in treatments T1, T2 and T4. In the case of treatment T3, which exhibited the highest biological activity, the composition is as follows: Ag_2_S acanthite (34.1%) and argentite (38.1%) NPs, AgNPs 5.8%, and AgCl 22.0%. In this case, the biological activity is attributed mainly to the Ag_2_S-S nanoparticles, since they are present in greater proportions, and to a lesser extent to the AgNPs.

The coexistence of these compartmentalized Ag_2_S–S Janus NPs with MT-AgNPs provides our formulations with unique physicochemical characteristics, particularly in terms of nanoparticle morphology, size, surface structure, and chemical composition. These structural features may, in turn, influence their interaction with fungal cells and contribute to the distinct antifungal activity observed in our formulations.

### 2.7. In Vitro Effect of Nanoparticles on Growth Progress and Mycelial Inhibition of Phytopathogens

Statistically significant differences (*p* < 0.001) were found in all treatments. It was observed that in all isolates, NPs T1 and T2 showed a better effect on mycelial growth, except for *Alternaria*, where all NPs inhibited growth completely. The monomolecular one was the best mathematical model that described mycelial growth’s temporal progress: y = 1−(1−y0) exp(−rMt). In the case of *P. capsici*, it was observed that T1 and T3 showed an inhibition percentage of 68.83 and 67.90%, respectively at the final day of evaluation (seven days). Likewise, these NPs delayed the mycelial growth of *Phytophthora* for two days, which was reflected in a final growth of 25.25 and 26 mm for T1 and T3, respectively. Furthermore, we found that the rate of increase was lower (0.056 and 0.059) than the other treatments. All this can be seen integratively in a smaller area under the curve, 103.69 for T1 and 113.81 for T3 (Table 3).

The assays conducted on *A. alternata* revealed a complete inhibition of mycelial growth in response to the tested NP treatments. As a result, it was not possible to estimate growth-related parameters or fit descriptive growth models. This finding has practical implications, suggesting the potential use of NPs in the integrated management of *Alternaria* in agricultural fields (Table 4).

In *Fusarium*, T1 and T3 NPs inhibited 54.55 and 54.63%, respectively at the final day of evaluation (12 days). Likewise, the time to start mycelial growth was two days. Mycelial growth at the end of the evaluation, the rate of increase, and the area under the curve were lower than in the other NPs treatments (Table 5).

In the case of *Colletotrichum*, the percentage of inhibition of T1 and T3 was 32.26 and 33.69% at the final day of evaluation (17 days), which is lower if we compare the effect on *Phytophthora* and *Fusarium*. In addition, the chemical control Captan 50^®^ resulted in 73.22% inhibition at day 7 (Figure 7 and Figure 8) and 24.65% at day 17 (Table 6), indicating that the effect of Captan 50^®^ on *C. boninense* is fungistatic. Likewise, the nanoparticles exhibited a fungistatic effect on the fungus, achieving inhibition in the 50–70% range after 7 days of evaluation and in the 23.66–33.69% range by the end of the study period.

However, in *Colletotrichum*, it was observed that NPs T1 and T3 increased the time to start mycelial growth, which was five days. Likewise, these NPs have an effect by reducing the rate of increase and the area under the curve and are considered the best NPs (Table 6).

The use of NPs in this research showed evidence of their effect on controlling phytopathogenic fungi and oomycetes, which is an innovation for the agricultural production system, since they are not toxic to human health and, due to their multisite effect, do not induce resistance in fungi. Furthermore, it could be seen that NPs T1 and T3 have a lower rate of increase and a lower area under the curve for *Phytophthora*. The time at the beginning of mycelial growth was longer in the case of *Colletotrichum*, which is very important in integrated crop management, since the more time passes for the fungus to grow, the greater the opportunity that the crop must overcome infection. This would be reflected in fewer applications for fungal control, which has an economic impact, since the expenditure of agricultural producers is reduced.

### 2.8. Mean Inhibitory Concentration (IC_50_) of Nanoparticles Against F. oxysporum f. sp. lycopersici

The IC_50_ value of nanoparticle formulation T3 against Fol was determined because of the economic importance and wide host range of this pathogen, which is considered one of the most destructive species within the genus *Fusarium* [40]. The IC_50_ value for T3 was 22.62 µg∙mL^−1^, whereas the commercial fungicide Captán 50^®^ exhibited an IC_50_ of 22.36 µg∙mL^−1^.

Although Captán 50^®^ showed slightly higher antifungal activity, the comparable IC_50_ values suggest that T3 NPs represent a competitive alternative for the management of *Fusarium* wilt, particularly considering their potential advantages in terms of reduced environmental impact and a lower risk of resistance development.

### 2.9. Effect of T3 Nanoparticles on Vascular Wilt Caused by Fol in Tomato

Only the Adonis F1 variety showed statistically significant differences (*p* = 0.001) for plant height at 10 days after treatment application. Healthy seedlings (treatments 1 and 2) and those treated with NPs (treatments 3 and 4) exhibited the greatest plant height (12.92–14.07 cm, *p* < 0.05) (Table 7). In contrast, no statistically significant differences in plant height were detected for variety 1 (7502 F1) across the evaluated treatments.

Overall, two distinct groups were observed among the treatments, separating Fol-inoculated from non-inoculated plants. The reduced plant height was therefore primarily associated with pathogen infection rather than nanoparticle application. These findings are consistent with previous reports indicating that exposure to AgNPs can stimulate early growth and biomass accumulation in healthy tomato seedlings [41].

The mean values for stem diameter and root length did not show statistically significant differences (*p* > 0.05) across treatments in any of the evaluations, indicating that NP application had no measurable effect on these parameters.

**Figure 10:**
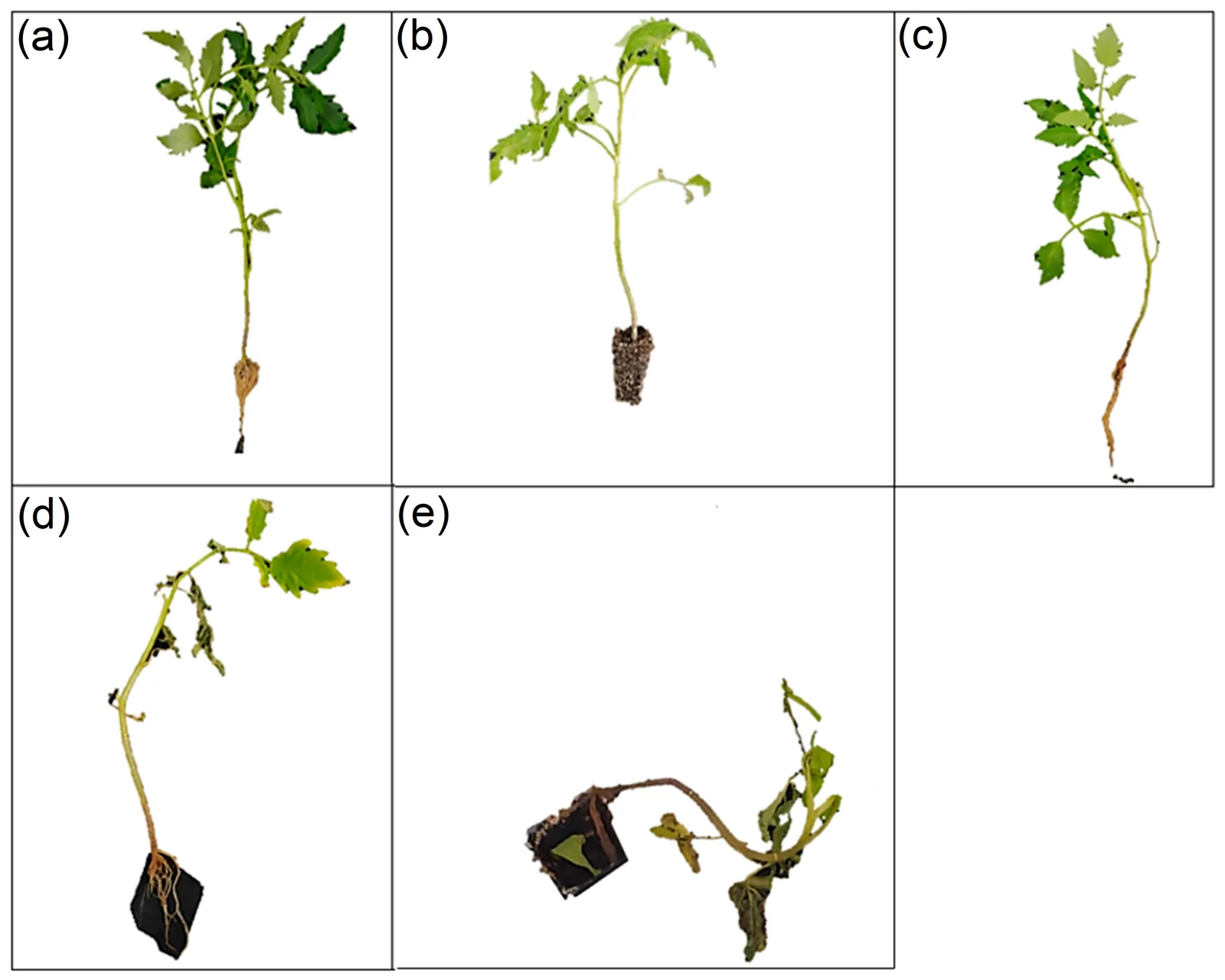
Symptoms observed in tomato seedlings infected with *Fusarium oxysporum* f. sp. *lycopersici*: (**a**) healthy seedling, (**b**) seedling with a whitish stem, (**c**) seedling with a brown stem and chlorosis, (**d**) seedling with a brown stem, chlorosis, and necrotic leaves, and (**e**) dead seedling.

Control seedlings and noninoculated treatments (treatments 1–4 and 9–12) did not exhibit any disease symptoms. In contrast, all Fol-inoculated treatments (treatments 5–8 and 13–16) developed symptoms within 72 h after inoculation. Disease incidence and severity were assessed using a symptom-based ordinal scale as follows: 0 = healthy seedling; 1 = seedling with whitish stem; 2 = seedling with brown stem and chlorosis; 3 = seedling with brown stem, chlorosis, and necrotic leaves; and 4 = dead seedling (Figure 10). The corresponding results are presented in Table S1.

**Figure 11:**
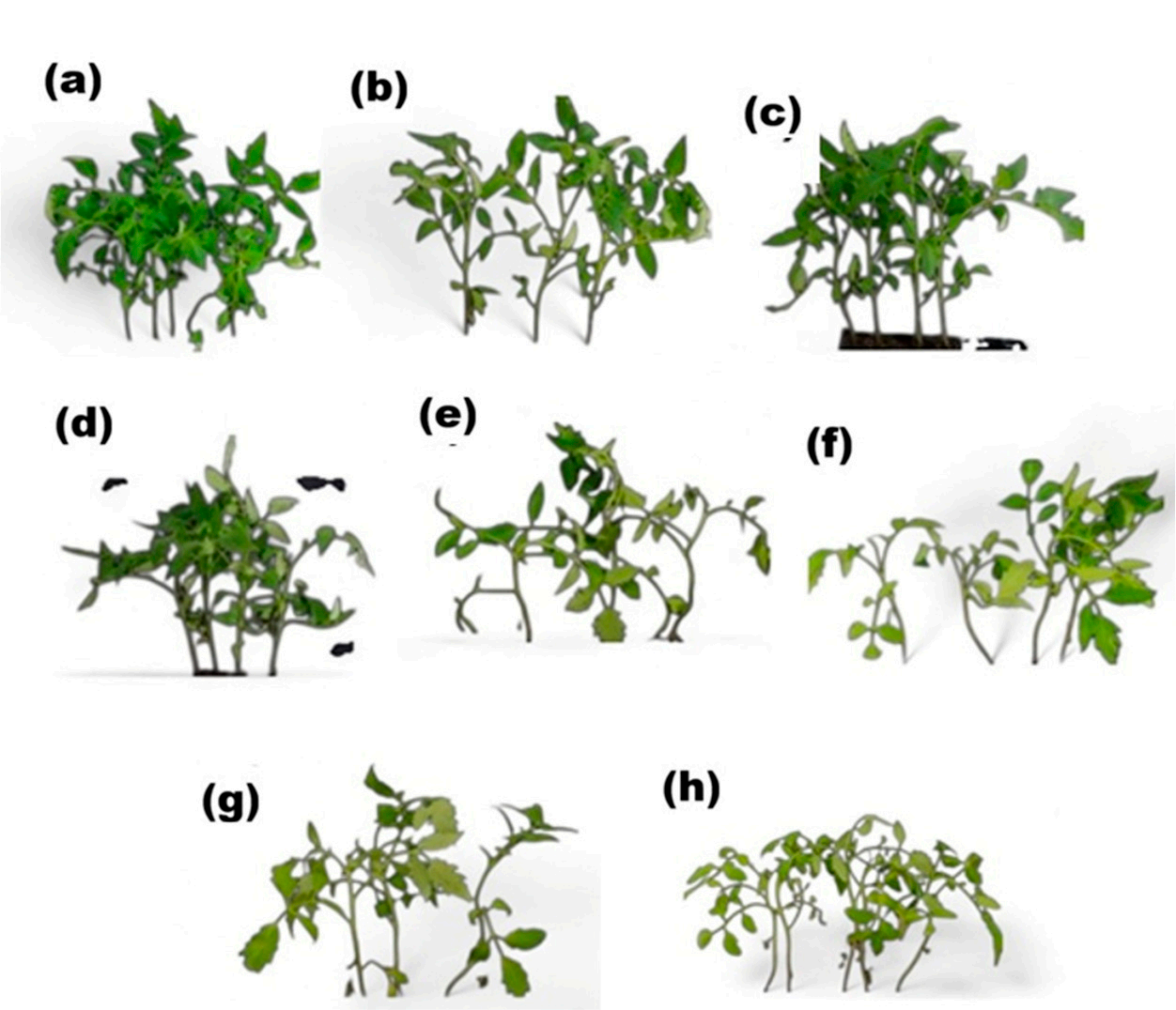
Tomato seedlings of the Adonis F1 variety under different treatments: (**a**) control, (**b**) control + wounding, (**c**) two applications of nanoparticles (NPs 2), (**d**) one application of nanoparticles (NPs 1), (**e**) *Fusarium oxysporum* f. sp. *lycopersici* (Fol), (**f**) Fol + NPs 2, (**g**) Fol + NPs 1, and (**h**) Fol + Captán 50^®^.

**Figure 12:**
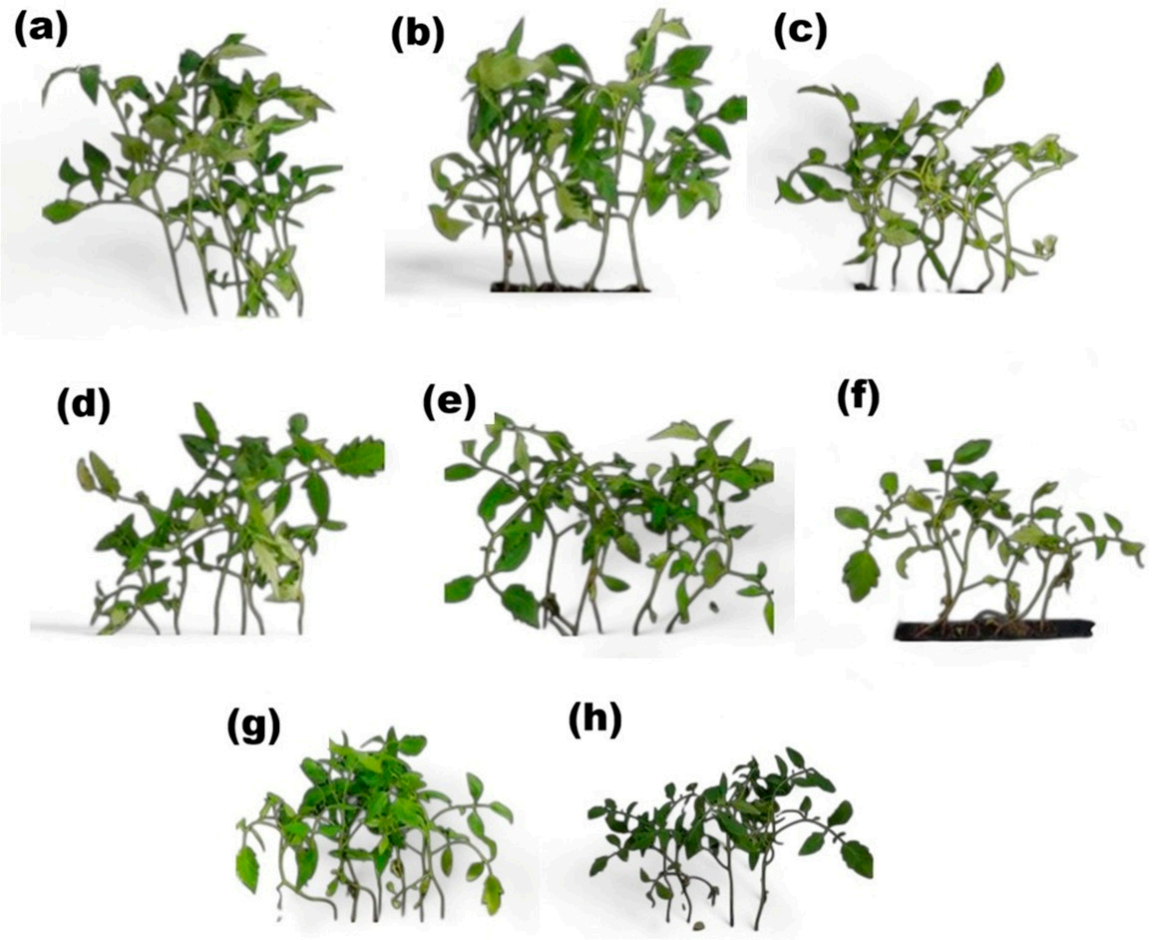
Tomato seedlings of the 7502 F1 variety under different treatments: (**a**) control, (**b**) control + wounding, (**c**) two applications of nanoparticles (NPs 2), (**d**) one application of nanoparticles (NPs 1), (**e**) *Fusarium oxysporum* f. sp. *lycopersici* (Fol), (**f**) Fol + NPs 2, (**g**) Fol + NPs 1, and (**h**) Fol + Captán 50^®^.

Figure 11 and Figure 12 illustrate the differences in leaf and stem coloration among treatments. Non-inoculated seedlings exhibited dark green leaves and stems. In contrast, seedlings inoculated with Fol and treated with NPs or Captán 50^®^ developed progressive foliar yellowing followed by necrosis, with some leaves remaining attached to the stem. In addition, inoculated seedlings displayed brown discoloration at the stem base, which is characteristic of vascular wilt symptoms [42]. Disease severity was lower in variety 1 (Adonis F1) than in variety 2 (7502 F1) (Table S1).

In both tomato varieties, seedlings inoculated with Fol and treated with NPs and Captán 50^®^ (treatments 6–8 and 14–16) showed gum and crust formation sealing the wound at the inoculation site 5 days after treatment application (severity scale 1–2, Table S1, Figure 11 and Figure 12). These results indicate that NP application effectively reduced disease development caused by Fol in tomato seedlings.

Previous studies have similarly reported that AgNPs decrease the incidence and severity of *Fusarium* wilt and reduce the number of wilted plants [43,44]. Likewise, Gomma et al. [44] demonstrated that AgNP application in two different tomato rootstocks significantly reduced disease incidence and severity while increasing shoot and root fresh and dry biomass compared with untreated controls.

In contrast, treatments receiving the chemical control Captán 50^®^ (treatments 8 and 16) exhibited leaf margin burn symptoms (severity scale 3; Table S1). Leaf edge burn has previously been described as a symptom of Captán 50^®^ phytotoxicity, along with leaf curling, chlorosis, wrinkling, necrosis, and reduced yield [45].

### 2.10. Nutritional Analysis of Tomato Seedlings

Tables S2–S4 present the concentrations of macroelements, microelements, and Ag in the root, stem, and leaf tissues of tomato seedlings. Previous studies have shown that plants exposed to AgNPs and metal oxides can accumulate these materials in the roots and subsequently translocate them to aerial tissues [18,46]. Ag translocation within plants varies depending on the chemical form and transformation of Ag, as well as whether Ag_2_S or Ag NPs are applied. AgNP exposure generally results in higher Ag accumulation in the roots of rice, lettuce, alfalfa, and wheat than Ag_2_S NP exposure. Moreover, Ag_2_S NPs showed lower translocation factors than AgNPs in wheat but higher values in rice, and were also translocated from roots to soybean leaves, where they accumulated [47]. In the present study, Ag accumulated predominantly in the roots and, to a lesser extent, in the stems (Tables S3 and S4), with no detectable translocation to the leaves (Table S2).

Regarding nutritional analysis, AgNPs have been reported to influence water and nutrient absorption by altering membrane fluidity and permeability in plant cells [48]. In this study, statistically significant correlations were detected only in stem tissue between Ca and Ag concentrations (r = −0.523, *p* = 0.038) and between Zn and Ag concentrations (r = 0.601, *p* = 0.014) (Table S5). The calcium concentration in the stem decreased, whereas the Zn concentration increased following NPs application. No statistically significant correlations were observed between Ag and the concentrations of N, P, K, Mg, Na, Fe, Mn, Cu, S, or B.

Similarly, Shams et al. [49] reported that foliar application of AgNPs (0–1000 µg∙mL^−1^; 35 nm) in tomato plants reduced the mineral concentrations of B, Zn, Ca, P, K, Fe, Cu, Mn, Na, and Mg in different tissues of healthy seedlings. An inverse relationship between Ag and Ca has also been reported in other crops. For example, exposure of radish plants to AgNPs (1–10 nm) at high concentrations (500 µg∙mL^−1^) reduced the accumulation of the macro-elements Ca and Mg [50].

Ag^+^ may interfere with cation-recognition sites at the plasma membrane, thereby disrupting Ca^2+^ influx. The resulting reduction in cytosolic Ca^2+^ availability may impair Ca^2+^ binding to calcium-binding proteins such as calmodulin, consequently preventing the activation of the HSF/HSP signaling cascade [51]. Ag^+^ has a high affinity for negatively charged sites in cell walls and membranes and can compete with or displace other cations. Moreover, Ag ions can interact with sulfhydryl-containing proteins and inhibit enzyme activity, potentially altering ion transport and cellular signaling. Ag may also interfere with Ca^2+^ influx without necessarily impairing metabolic activity or the electrochemical gradients that drive ion transport. Consistent with this mechanism, increasing Ag^+^ concentrations have been reported to decrease Ca content in the shoots while increasing its accumulation in the roots of bean plants [18,52,53].

However, in this study, the precise chemical form of Ag within tomato plant tissues—whether ionic Ag^+^, Ag_2_S, or metallic Ag^0^—remains uncertain, and its bioavailability may depend on its transformation and interaction with cellular components. The reduction in Ca concentration observed at 1000 µg∙mL^−1^ of NPs should not be attributed exclusively to direct inhibition of calcium channels. Rather, it may reflect a combination of altered cation recognition and transport, competition between Ag and Ca^2+^ at membrane-associated binding sites, and changes in the physicochemical properties of Ag within plant tissues.

## 3. Materials and Methods

### 3.1. Nanoparticle Synthesis

The Ag_2_S-S and AgNPs were prepared using silver nitrate (Sigma-Aldrich, St. Louis, MO, USA) as a precursor, glucose (C_6_H_12_O_6_) as a reducing agent, gelatin (Sigma-Aldrich, St. Louis, MO, USA) as a stabilizer, NaOH (JTBaker^®^, Phillipsburg, NJ, USA) to adjust the pH, and deionized water (18 MΩ∙cm^−1^, Easypure) as a solvent.

The Ag_2_S-S and AgNPs were synthesized according to the methodology proposed by Aguilar-Méndez et al. [25], with some modifications. According to the concentrations (Table 8), aqueous solutions of gelatin and glucose were prepared in 25 mL of deionized water; the solution was adjusted to a pH of 10 by adding NaOH. Solution was placed in a flask under magnetic stirring and temperature control. When the solution reached 80 °C an aqueous solution of 0.1 M AgNO_3_ was added drop by drop subsequently, the solution acquired a dark brown color, indicating the formation of Ag_2_S-S and AgNPs [26]. The solution was maintained for another 15 min under the same temperature and stirring conditions. The resulting colloidal Ag_2_S-S and AgNPs solution was cooled to room temperature.

The NPs obtained may result from the following reactions:

(1) 2Ag^+^ + 2OH^−^ → Ag_2_O + H_2_O

(2) 2Ag_2_O + C_6_ H_12_ O_6_ + S^2−^(Gelatin) → C_6_H_12_O_7_ + 2Ag^0^ (AgNPs) +Ag_2_S (Ag_2_S NPs) + Gelatin

### 3.2. Nanoparticle Characterization

The UV-Visible spectra of the nanoparticles were obtained in a spectrophotometer (Thermo Scientific Multiskan-Go, Vantaa, Finland) in an absorbance range of 300–550 nm. The crystal structure of the samples was characterized by X-ray diffraction (XRD), performed with a diffractometer Bruker D8 ADVANCE ECO (Bruker, Karlsruhe, Germany), using Cu-Kα radiation (λ = 1.5418 Å) as the X-ray source in a 2θ range of 20–80°. The morphology, size, and elemental composition were determined by transmission electron microscopy (TEM) in a Jeol ARM200F microscope (JEOL Ltd., Tokyo, Japan) operated at 200 kV (LUME@UNAM; RRID SCR_022400) and coupled with an Oxford AZtecMTE detector for energy dispersive X-ray spectroscopy (EDS, Plano, TX, USA). For analysis, a drop of the Ag_2_S-S and AgNPs was dispersed in acetone with deionized water (1:4) and sonicated for 5 min; subsequently, a drop was placed on a carbon-coated copper grid and finally observed in the electron microscope. The hydrodynamic diameter, polydispersity index (PDI), and zeta potential of the synthesized NPs were determined using a Zeta Sizer Nano ZS ZEN 3600 (Malvern Instruments, Malvern, UK). The NP samples were diluted with deionized water and sonicated prior to analysis to achieve a uniform dispersion. Measurements were performed at 25 ± 2 °C.

### 3.3. In Vitro Inhibition of Plant Pathogens

The antifungal and anti-oomycete activity was determined by evaluating the effect of the four NPs synthesized by the agar dilution method of Sánchez-Fernández et al. [54] on the radial growth of three plant pathogenic fungi, *A. alternata*, *F. oxysporum*, and *C. boninense*, and the oomycete *P. capsici*. The Mycology Laboratory of the Department of Agricultural Parasitology of the Universidad Autónoma Chapingo provided the microorganisms.

NPs were dispersed in deionized sterilized water and added to potato dextrose agar (PDA, BD BioxonTM) (1:1) to a final 100 μg ∙mL^−1^ concentration. The evaluation was performed using 10 cm diameter Petri dishes, where each NP was placed and allowed to completely solidify. Then, a 0.6 cm diameter inoculum from the mycelium of each plant pathogenic fungi and oomycete culture on PDA was placed in the center of each plate and incubated at 28 °C, under a 12:12 h fluorescent light-dark photoperiod for 20 days. As positive controls, two commercial fungicides were used at 100 μg∙mL^−1^: Captán 50^®^ (*N*-trichloromethylthio-cyclohex-4-en-1,2-dicarboximide, 50% *w*/*w*, ADAMA, Airport City, Israel) for fungi, and Ridomil Gold^®^ 480 SL (metalaxyl-M: Methyl N-(methoxyacetyl)-N-(2,6-xylyl)-D-alaninate, 45.28% *w*/*w*, Syngenta, Basel, Switzerland) for the oomycete *P. capsici*. The negative control was a solution of PDA-water (1:1). Bioassays were performed under a completely randomized design with four repetitions. Every 24 h, mycelial growth was measured with the help of a digital vernier (Truper ^®^) for *Phytophthora*, *Alternaria*, *Fusarium*, and *Colletotrichum*, subjected to the six treatments. The measurements were completed until the absolute control of each isolate filled the Petri dish, specifically, the incubation periods were seven days for *P. capsici*, nine days for *A. alternata*, 12 days for *F. oxysporum*, and 17 days for *C. boninense* [54].

Based on the data on mycelial growth, it was determined which mathematical model (logistic, monomolecular, exponential, Gompertz) described the temporal progress of the growth of each of the isolates in the different treatments to calculate the growth rate parameter (rM). The time at the beginning of mycelial growth was recorded in days (X_0_), as well as the final growth at the end of the evaluation (yF), and as an integrative parameter of the temporal analysis of growth, the area under the mycelial growth curve was calculated (ABCCM) with the trapezoidal integration method [55]. To measure the efficiency of each NPs type, the percentage of inhibition was calculated with the Abbott (1925) formula [56].

To analyze the effect of the parameters mentioned on the treatments, an analysis of variance (ANOVA, α = 0.05) was carried out, as well as a comparison of means using the LSD test (α = 0.05) in SAS OnDemand for Academics software (v. 9)^®^.

### 3.4. Mean Inhibitory Concentration (IC_50_) on F. oxysporum f. sp. lycopersici

The IC_50_ values of the antifungal nanoparticle formulation T3 (10 to 150 μg∙mL^−1^) and the commercial fungicide Captán 50^®^ (1 to 50 μg∙mL^−1^ [3.33 to 166.34 µM]) were determined based on the radial growth inhibition of Fol using the agar dilution method. The percentage of mycelial growth inhibition was calculated for each concentration.

The Fol isolate was provided by the Mycology Laboratory of the Department of Agricultural Parasitology of the Universidad Autónoma Chapingo. Bioactivity data were statistically analyzed, and IC_50_ values were estimated using the SAS OnDemand for Academics software.

### 3.5. Evaluation of Nanoparticle Effects on Vascular Wilt Caused by Fusarium oxysporum f. sp. lycopersici in Tomato

The effect of nanoparticle formulation T3 on vascular wilt caused by Fol in tomato was evaluated at 1000 μg∙mL^−1^. The experiment was conducted using a completely randomized design comprising six treatments, including two controls, two tomato varieties, and four replicates per treatment (Table 9). Captán 50^®^ was included as a positive control at a concentration of 1000 μg∙mL^−1^.

Two tomato (*Solanum lycopersicum*) varieties, Adonis F1 and 7502 F1, were used in this study. Seedlings were established in germination trays containing peat moss as substrate, which was sterilized by autoclaving at 120 °C for 20 min. Trays were disinfected with 2% NaClO solution and rinsed with distilled water. Seeds were immersed in 70% ethanol for 1 min and subsequently rinsed four times with distilled water. Sowing was performed at a depth of 5 mm. Seedlings were fertilized with 50% Steiner nutrient solution after the emergence of the second true leaf.

For inoculation, 15-day-old cultures of Fol grown on Petri dishes were used, following the methodology of Cardona and Castaño [57], with minor modifications. Seedlings inoculated (treatments 5–8 and 13–16; Table 9) were 50 days old. A superficial wound was made at the base of the stem using a sterile syringe, and a 1 cm-diameter mycelial plug of Fol was placed directly onto the tissue. The inoculation site was sealed with Parafilm^®^. Additionally, Parafilm^®^ was placed beneath the tray cavities to prevent leakage. Trays were enclosed in transparent plastic bags, misted with distilled water to maintain high humidity, and sealed for 24 h.

Nanoparticle formulation T3 was applied to the substrate at a dose of 5 mL per seedling (1000 μg∙mL^−1^). Treatments 3, 6, 11, and 14 received two applications of T3 (Table 9). In treatments 6 and 14, the first application was performed before Fol inoculation, followed by inoculation, and a second application was made 24 h later. Treatments 4, 7, 12, and 15 received a single application; in treatments 7 and 15, NPs were applied after pathogen inoculation. Captán 50^®^ was applied once to the substrate of Fol-inoculated seedlings (treatments 8 and 16) at 5 mL per seedling (1000 μg∙mL^−1^). Seedlings were maintained under natural sunlight conditions and irrigated every 2–3 days as required.

To assess the protective effect of T3 NPs against Fol-induced vascular wilt, as well as potential phytotoxicity, the following variables were measured: plant height, stem diameter, root length, disease incidence and severity, absorption of macro- and micronutrients, and Ag accumulation.

### 3.6. Nutritional Analysis and Silver Absorption

For nutrient analysis, seedling roots were washed with distilled water, and plant tissues (leaves, stems, and roots) were separated. Samples were freeze-dried and subsequently subjected to wet digestion. Nitric acid digestion was used for the determination of P, K, S, Ca, Mg, Na, B, Zn, Mn, Fe, Cu, Mo, and Ag, whereas sulfuric acid digestion was employed for total nitrogen [41]. Total nitrogen was quantified using a flow injection analyzer (QuikChem^®^ 8500 Series 2, Lachat, Loveland, CO, USA). P, S, and B were determined colorimetrically using a UV-Vis spectrophotometer (Jenway^®^ 6715, Stone, UK). Na and K were measured by flame photometry (QuikChem^®^ Lachat, Loveland, CO, USA). Ca, Mg, Co, Fe, Mn, Zn, and Ag were quantified using atomic absorption spectrophotometry (Ultima 2, Horiba Jobin Yvon Inc., Austin, TX, USA) [58,59,60].

### 3.7. Statistical Analysis

Data were analyzed using one-way analysis of variance (ANOVA), followed by Tukey’s test for mean comparison at a 95% confidence level (α = 0.05). In addition, multiple correlation analysis was performed to evaluate relationships among macro- and micronutrient concentrations and Ag content, and Pearson correlation coefficients were calculated. All analyses were performed using SAS OnDemand for Academics software.

## 4. Conclusions

These results highlight the potential of Ag_2_S-S Janus and Ag NPs (T1–T4) as antimicrobial agents for the control of diseases caused by phytopathogenic fungi and oomycetes. Among the evaluated formulations, the smallest NPs (T1-7.79 nm and T3-5.9 nm) exhibited the highest antifungal and anti-oomycete activity, highlighting the influence of nanoparticle size on biological efficacy.

Formulation T3 effectively inhibited the growth of the tested phytopathogenic fungi and oomycetes and showed promising activity against *Fusarium oxysporum* f. sp. *lycopersici*, the causal agent of tomato vascular wilt. Application of T3 reduced disease severity in inoculated seedlings to levels corresponding to severity scale values of 1–2, indicating partial suppression of disease development.

Only variety 1 (Adonis F1) showed significant differences in seedling height following treatment application. However, NP application at 1000 μg∙mL^−1^ did not negatively affect the overall growth of tomato seedlings infected with Fol, suggesting low phytotoxicity under the evaluated conditions. In addition, NP treatment influenced Ca and Zn absorption in stem tissues of Fol-inoculated seedlings, indicating that Ag-based NPs may alter nutrient dynamics in tomato plants.

Overall, these findings support the potential use of Ag-based NPs as alternative tools for the management of *Fusarium* wilt in tomato. Nevertheless, further in vitro, greenhouse, and field studies are required to optimize NP formulations, evaluate their long-term effectiveness, and assess their environmental safety and agronomic applicability under commercial production conditions.

## Figures and Tables

**Table 1:** XRD crystal size results.

Sample	Size Ag (nm)	Size Ag_2_S (nm)
T1	14.31	26.34
T2	9.52	20.58
T3	1.92	25.65
T4	15.33	16.11

**Table 2:** Hydrodynamic diameter (HD), polydispersity index (PDI), and Z-potential values for the synthesized Janus Ag_2_S-S and Ag nanoparticles.

Treatment	HD	PDI	Z-Potential
T1	137 ± 0.5	0.22 ± 0.06	−18 ± 4.7
T2	402 ± 16	0.38 ± 0.03	−16 ± 3.0
T3	131 ± 24	0.19 ± 0.01	−23 ± 2.8
T4	602 ± 51	0.51 ± 0.05	−8 ± 2.7

**Table 3:** Effect of nanoparticles on the growth of *Phytophthora capsici*.

Treatment	Inhibition (%)	X_0_	yF	rM (mm∙h^−1^)	ABCCM (mm∙h^−1^)	R^2^	SE
PDA	0 d	1	81 a	0.30227 a	356.19 a	0.98	0.0117
Chemical control	100 a	>7	0 d	0 d	0 d	nd	nd
T1	68.827 b	2	25.250 c	0.05670 c	103.69 c	0.95	0.00510
T2	58.796 c	2	33.375 b	0.07820 b	134.69 b	0.98	0.05580
T3	67.90 b	2	26.00 c	0.05965 c	113.81 c	0.96	0.06275
T4	60.80 c	2	31.75 b	0.07599 b	135.06 b	0.98	0.05138

X_0_: time to start mycelial growth; yF: final growth in mm; rM: monomolecular model rate parameter; ABCCM: area under the mycelial growth curve. R^2^: coefficient of determination; SE: standard error. Different letters indicate statistically significant differences between treatments (Fisher, *p* < 0.05).

**Table 4:** Effect of nanoparticles on the growth of *Alternaria alternata*.

Treatment	Inhibition (%)	X_0_	yF	rM (mm∙h^−1^)	ABCCM (mm∙h^−1^)	R^2^	SE
PDA	0 b	1	81 a	0.6029628 a	399.875 a	0.95	0.07225
Chemical control	100 a	>9	0 b	0 b	0 b	nd	nd
T1	100 a	>9	0 b	0 b	0 b	nd	nd
T2	100 a	>9	0 b	0 b	0 b	nd	nd
T3	100 a	>9	0 b	0 b	0 b	nd	nd
T4	100 a	>9	0 b	0 b	0 b	nd	nd

X_0_: time to start mycelial growth; yF: final growth in mm; rM: monomolecular model rate parameter; ABCCM: area under the mycelial growth curve. R^2^: coefficient of determination; SE: standard error. Different letters indicate statistically significant differences between treatments (Fisher, *p* < 0.05).

**Table 5:** Effect of nanoparticles on the growth of *Fusarium oxysporum*.

Treatment	Inhibition (%)	X_0_	yF	rM (mm∙h^−1^)	ABCCM (mm∙h^−1^)	R^2^	SE
PDA	0 e	1	81 a	0.41824 a	588.75 a	0.97	0.1217
Chemical control	100 a	>12	0 e	0 e	0 f	nd	nd
T1	54.552 b	2	36.813 d	0.05015 d	264.25 d	0.97	0.0392
T2	31.33 d	3	55.625 b	0.09007 b	379.56 b	0.98	0.0664
T3	54.63 b	2	36.75 d	0.05218 d	216.9 e	0.97	0.0458
T4	39.583 c	2	48.938 c	0.07367 c	331.13 c	0.98	0.0517

X_0_: time to start mycelial growth; yF: final growth in mm; rM: monomolecular model rate parameter; ABCCM: area under the mycelial growth curve. R^2^: coefficient of determination; SE: standard error. Different letters indicate statistically significant differences between treatments (Fisher, *p* < 0.05).

**Table 6:** Effect of nanoparticles on the growth of *Colletotrichum boninense*.

Treatment	Inhibition (%)	X_0_	yF	rM (mm∙h^−1^)	ABCCM (mm∙h^−1^)	R^2^	SE
PDA	0 c	2	74.5 a	0.3585 a	980.19 a	0.98	0.08440
Chemical control	24.657 b	7	56.125 b	0.1935 b	453.63 c	0.94	0.04053
T1	32.26 a	5	50.44 c	0.05134 c	459.1 c	0.97	0.03211
T2	23.63 b	4	56.875 b	0.06457 c	567.63 b	0.98	0.04426
T3	33.69 a	5	49.38 c	0.04916 c	445.5 c	0.97	0.03352
T4	26.98 b	3	54.38 b	0.05625 c	532.7 b	0.98	0.03652

X_0_: time to start of mycelial growth; yF: final growth in mm; rM: monomolecular model rate parameter; ABCCM: area under the mycelial growth curve. R^2^: coefficient of determination; SE: standard error. Different letters indicate statistically significant differences between treatments (Fisher, *p* < 0.05).

**Table 7:** Mean comparison of plant height in Adonis F1 tomato seedlings at 10 days after treatment application.

Identification	Treatments	Height (cm)
1	Ctrl	12.92 ± 1.97 a
2	Ctrl + H	13.83 ± 0.92 a
3	NPs 2	14.00 ± 1.46 a
4	NPs 1	14.07 ± 1.40 a
5	F	10.30 ± 0.65 c
6	F NPs 2	9.92 ± 1.13 c
7	F NPs 1	10.83 ± 0.86 bc
8	F + Captán 50^®^	12.38 ± 1.13 ab

Mean ± standard deviation. Different letters indicate statistically significant differences between treatments (Tukey, *p* ≤ 0.05).

**Table 8:** Glucose and gelatin concentrations employed for the synthesis of Ag_2_S-S Janus and Ag nanoparticles.

Treatment	Glucose Concentration (%)	Gelatin Concentration (%)
T1	0.30	0.50
T2	0.30	1.00
T3	0.90	0.50
T4	0.90	1.00

**Table 9:** Description of the treatments evaluated.

Identification	Variety *	Treatment	Description
1	1	Ctrl	Healthy seedlings
2	1	Ctrl + W	Healthy seedlings with stem wounding
3	1	NPs 2	Healthy seedlings + 5 mL of NPs (two applications)
4	1	NPs 1	Healthy seedlings + 5 mL of NPs (one application)
5	1	F	Seedlings inoculated with Fol
6	1	F + NPs 2	Seedlings inoculated with Fol + NPs (two applications)
7	1	F + NPs 1	Seedlings inoculated with Fol + NPs (one application)
8	1	F + Captán 50^®^	Seedlings inoculated with Fol + Captán^®^
9	2	Ctrl	Healthy seedlings
10	2	Ctrl + W	Healthy seedlings with stem wounding
11	2	NPs 2	Healthy seedlings + 5 mL of NPs (two applications)
12	2	NPs 1	Healthy seedlings + 5 mL of NPs (one application)
13	2	F	Seedlings inoculated with Fol
14	2	F + NPs 2	Seedlings inoculated with Fol + NPs (two applications)
15	2	F + NPs 1	Seedlings inoculated with Fol + NPs (one application)
16	2	F + Captán 50^®^	Seedlings inoculated with Fol + Captán^®^

* Variety 1: Adonis F1 tomato; variety 2: 7502 F1 tomato. Two applications: before and after inoculation with Fol; one application: after inoculation with Fol.

## Data Availability

The original contributions presented in this study are included in the article. Further inquiries can be directed to the corresponding author.

## Supplementary Materials

The following supporting information can be downloaded at https://www.mdpi.com/article/10.3390/ijms27177916/s1.

## Abbreviations

NPs	Nanoparticles
ROS	Reactive oxygen species
HRTEM	High-Resolution Transmission Electron Microscopy
HAADF	High-Angle Annular Dark-field
FFT	Fast Fourier transform
EDS	Energy-dispersive spectroscopy
SD	Standard deviation
