# Supplementary material for: Antifungal and Anti-Oomycete Potential of Ag2S-S Janus and Ag Nanoparticles Synthesized with Non-Toxic Agents and Their Application to Control Fusarium Wilt of Tomato
Source: Int J Mol Sci. 2026 Sep 4;27(17):7916. doi: 10.3390/ijms27177916 (PMC13565788; doi:10.3390/ijms27177916)
Supplement: Supplementary file 1 [file ijms-27-07916-s001.zip › ijms-4486330-supplementary.pdf]

Antifungal and anti-oomycete potential of Ag_2S-S Janus and Ag nanoparticles synthesized with non-toxic agents and their application to control *Fusarium* wilt of tomato.

Rosa Elvira Sánchez Fernández [1], Moisés Camacho Tapia [2], Daniel Canseco-González [3] , Guadalupe Stefanny Aguilar-Moreno [4], Miguel A. Aguilar-Méndez [5], Francisco Ascencio [1*], Leticia Rojas-Sandoval [6] and Elizabeth Navarro Cerón [1*].

[1] Laboratorio Nacional de Investigación y Servicio Agroalimentario y Forestal (LANISAF), Universidad Autónoma Chapingo. Carretera México-Texcoco km 38.5, Chapingo, Texcoco, Estado de México, C. P. 56230, México.; resf2012@gmail.com.

[2] Universidad Autónoma Chapingo, Departamento de Parasitología. Carretera México-Texcoco km 38.5, Chapingo, Texcoco, Estado de México, C. P. 56230, México.; moises.camachotapia@gmail.com.

[3] SECIHTI-Laboratorio Nacional de Investigación y Servicio Agroalimentario y Forestal. Universidad Autónoma Chapingo Texcoco de Mora, CP 56230. México. jdcanseco@secihti.mx.

[4] Secretaría de Ciencia, Humanidades, Tecnología e Innovación (SECIHTI) - Universidad Autónoma Chapingo. Carretera México-Texcoco km 38.5, Chapingo, Texcoco, Estado de México, C. P. 56230, México.; stefanny.aguilar.m@gmail.com

[5] Instituto Politécnico Nacional, Centro de Investigación en Ciencia Aplicada y Tecnologíia Avanzada, Legaria 694, Colonia Irrigación 11500 México, DF, México.

[6] Universidad Autónoma Chapingo, Departamento de Fitotecnia, Carretera México-Texcoco km 38.5, Chapingo, Texcoco, Estado de México, C. P. 56230, México.; letizia1234rojaz@gmail.com.

*Corresponding author: h90018@chapingo.mx (E.N.C.); h90003@chapingo.mx (F.A.).

Table S1. Description of expressed characteristics of healthy tomato seedlings and seedlings infected with Fol and treated with NPs and Captan.

**Treatment**	**Description of symptoms**	**Severity scale**	**Incidence**
**1**	Green leaves, vigorous seedling growth, defoliation of the first two leaves (Figure 10 a)	0	0
**2**	Green leaves, vigorous seedling growth, 2-3 defoliated leaves, callus at the base of the stem (Figure 10 b).	0	0
**3**	Green leaves, leaf 1 - 2 defoliated (Figure 10 c).	0	0
**4**	Green leaves, less stem elongation (Figure 10 d).	0	0
**5**	Leaves with yellowing in veins, drying of the 3-4 basal leaves (Figure 10 e).	1 - 3	100
**6**	Leaves with yellowing in veins, necrosis of the 2-3 basal leaves, brown coloration at the neck of the stem (Figure 10 f).	1 - 2	100
**7**	Leaves with yellowing in veins, the 3 basal leaves dried up, brown color in the neck of the stem (Figure 10 g).	1 - 2	100
**8**	Yellowing of the leaves, necrotic edges, the first 3 basal leaves dried up (Figure 10 h).	1 - 3	100
**9**	Green leaves, vigorous seedling growth (Figure 11 a).	0	0

**10**	Green leaves, defoliation of the basal 1-2 leaves (Figure 11 b).	0	0
**11**	Green leaves, slow growth, resulting in shorter seedlings (Figure 11 c).	0	0
**12**	Green leaves, defoliation of the first 1-2 basal leaves (Figure 11 d).	0	0
**13**	Yellowing of the leaves, necrosis of 3 basal leaves and their retention on the stem. A dead seedling, first showing brown color in the stem (Figure 11 e).	1 - 4	100
**14**	Slight chlorosis in leaves progressing upwards; the basal leaves 1-3 later became necrotic and remained attached to the stem. One seedling died; the first symptom was browning of the stem. The stem then became necrotic in an upward direction (Figure 11 f).	1 - 2	100
**15**	Moderate chlorosis in leaves, ascending necrosis of the first 4 basal leaves, which remained attached to the stem. The stem collar was light brown (Figure 11 g).	1 – 2	100
**16**	They showed burns on the edges of the leaves, and the basal 1-2 leaves were defoliated (Figure 11 h).	1-3	100

Nutritional analysis

Tables S2, S3 and S4 show the results of the quantification of minerals (macro- and micronutrients) and silver in the stem, foliage and root tissues of healthy tomato seedlings exposed to different treatments.

Table S2. Concentration of macroelements, microelements and silver in tomato seedling foliage.

Identification	N (%)	P (%)	K (%)	Na (%)	Ca (%)	Mg (%)	S (%)	Cu (mg Kg^{-1})	Fe (mg Kg^{-1})	Mn (mg Kg^{-1})	Zn (mg Kg^{-1})	B (mg Kg^{-1})	Ag (mg Kg^{-1})
**1**	0.10	0.84	6.76	0.90	3.63	0.99	1.58	48.51	289.11	178.22	54.65	43.56	0.00
**2**	2.86	0.79	6.87	0.62	4.01	0.97	0.65	1.48	172.75	63.18	0.15	41.95	0.00
**2**	2.70	0.79	6.71	0.65	3.85	0.94	1.28	2.96	135.11	63.61	7.69	30.57	0.00
**3**	2.90	0.79	6.89	0.69	4.33	1.03	1.60	23.31	86.98	51.09	69.10	58.53	0.00
**4**	2.83	0.74	6.42	0.74	4.29	1.01	1.92	13.89	220.73	33.10	30.43	42.49	0.00
**5**	3.18	0.69	7.03	0.59	2.54	0.67	0.72	41.91	234.71	118.34	50.69	42.41	0.00
**6**	3.40	0.84	3.40	1.05	3.38	0.83	1.77	2.98	195.44	188.49	26.54	99.21	0.00
**7**	3.66	0.85	7.76	0.95	3.16	0.70	1.23	9.48	230.54	222.55	58.73	81.84	0.00
**8**	3.55	0.80	7.83	0.88	3.23	0.76	0.45	13.46	150.55	141.58	46.41	39.88	0.00
**9**	3.16	1.00	7.76	0.74	4.37	1.14	0.61	7.49	194.11	115.27	1.35	41.42	0.00
**10**	3.06	0.84	7.76	0.71	4.12	0.97	0.40	14.93	130.85	133.89	64.87	26.19	0.00
**11**	3.37	0.94	8.73	0.83	3.64	0.79	1.68	35.43	77.84	233.93	52.37	61.82	0.00
**12**	3.56	0.94	6.96	0.64	3.89	0.92	0.88	19.31	58.85	243.07	63.27	47.52	0.00
**13**	2.90	0.94	8.31	0.77	3.61	0.88	0.82	13.43	185.83	186.32	29.88	32.71	0.00
**14**	2.57	0.80	7.28	0.17	3.57	0.87	1.49	9.94	176.94	154.57	47.32	82.01	0.00
**15**	3.39	0.85	6.19	0.87	3.73	0.86	1.35	6.49	261.24	134.87	65.93	64.94	0.00
**16**	4.37	1.10	7.09	0.76	3.54	0.91	0.46	8.99	136.86	47.45	22.63	64.94	0.00

Table S3. Concentration of macroelements, microelements and silver in the stem of tomato seedlings.

Identification	N (%)	P (%)	K (%)	Na (%)	Ca (%)	Mg (%)	S (%)	Cu (mg Kg^{-1})	Fe (mg Kg^{-1})	Mn (mg Kg^{-1})	Zn (mg Kg^{-1})	B (mg Kg^{-1})	Ag (mg Kg^{-1})
**1**	1.10	0.70	11.59	0.89	1.38	0.73	0.22	17.95	180.46	35.89	71.59	21.93	0.00
**2**	0.54	0.59	10.05	0.61	1.56	0.62	0.49	0.98	60.29	17.16	29.95	20.59	6.37
**3**	1.31	0.55	10.37	0.68	1.11	0.48	0.09	18.44	35.89	47.36	63.66	8.47	3.49
**4**	1.06	0.52	9.31	0.65	1.18	0.48	0.26	13.87	38.98	33.53	90.91	11.55	22.35
**5**	1.28	0.56	14.33	0.71	1.38	0.30	0.91	14.98	44.94	10.30	47.75	66.48	0.00
**6**	1.25	0.78	11.15	1.06	1.16	0.45	1.22	24.80	84.30	5.81	100.58	34.88	24.22
**7**	1.25	0.50	12.71	0.96	1.38	0.32	0.69	4.25	73.65	52.41	123.09	58.78	25.50
**8**	1.26	0.70	14.35	1.01	1.52	0.49	0.30	6.99	112.77	72.36	118.56	7.49	0.00
**9**	5.66	0.53	12.50	0.57	1.43	0.57	0.48	5.83	48.06	24.76	81.94	43.69	0.00
**10**	1.36	0.42	8.60	0.56	1.30	0.50	0.28	35.29	83.33	19.39	90.55	45.49	23.62
**11**	1.85	0.68	10.49	0.63	1.42	0.54	0.29	15.29	96.67	92.82	117.64	37.34	13.87
**12**	1.98	0.58	11.36	0.55	1.28	0.52	0.31	17.83	70.72	70.90	109.20	48.92	22.80
**13**	1.08	0.73	13.26	0.70	1.43	0.52	0.48	21.78	77.79	57.73	54.16	19.08	0.00
**14**	1.05	0.55	11.93	0.87	1.22	0.49	0.42	4.98	56.27	42.33	102.49	397.41	17.43
**15**	1.71	0.65	11.59	0.80	1.48	0.56	0.31	53.95	60.44	31.97	51.05	15.98	6.49
**16**	0.52	0.60	10.85	0.87	1.43	0.52	0.36	47.00	69.00	50.00	61.65	0.00	0.00

Table S4. Concentration of macroelements, microelements and silver in the root of tomato seedlings.

Identification	N (%)	P (%)	K (%)	Na (%)	Ca (%)	Mg (%)	S (%)	Cu (mg Kg^{-1})	Fe (mg Kg^{-1})	Mn (mg Kg^{-1})	Zn (mg Kg^{-1})	B (mg Kg^{-1})	Ag (mg Kg^{-1})
**1**	1.91	0.65	2.64	0.92	0.81	0.40	0.72	53.23	317.74	184.68	56.29	83.06	3.23
**2**	0.83	0.56	2.94	0.82	0.80	0.38	0.88	23.41	194.76	41.20	59.08	35.58	0.00
**3**	1.58	0.55	4.02	0.58	0.91	0.39	0.18	16.47	169.16	52.89	138.72	39.42	224.05
**4**	2.69	0.46	3.39	0.63	0.74	0.30	0.39	33.53	283.27	39.74	85.89	38.49	141.63
**5**	2.22	0.08	0.44	0.05	0.11	0.12	0.27	9.09	37.12	15.53	9.85	18.94	8.68
**6**	1.17	0.71	2.67	0.50	0.73	0.04	1.38	10.66	197.87	105.45	187.91	16.59	302.63
**7**	1.89	0.50	3.00	0.48	0.74	0.85	1.75	43.05	235.10	198.68	305.46	18.21	274.83
**8**	2.22	0.55	5.11	0.68	0.82	0.30	0.69	32.24	191.47	203.87	122.57	300.60	0.00
**9**	1.53	0.59	3.11	0.82	0.61	0.35	1.03	34.04	431.92	100.94	30.75	99.77	0.00
**10**	2.18	0.50	4.31	0.75	0.79	0.48	0.38	33.33	167.32	217.78	127.72	13.89	82.22
**11**	2.81	0.62	2.98	0.55	0.80	0.21	0.82	51.12	207.35	528.68	295.32	324.19	69.20
**12**	2.01	0.60	4.90	0.64	0.79	0.26	0.45	41.75	301.28	60.14	236.93	16.90	120.28
**13**	1.48	0.87	3.34	0.92	0.66	0.29	0.91	36.54	184.62	336.54	25.96	54.81	0.00
**14**	3.35	0.49	2.71	0.73	0.77	0.12	0.86	27.96	160.36	157.07	189.80	18.91	216.28
**15**	1.07	0.46	3.20	0.71	0.77	0.24	0.57	33.21	142.50	152.86	150.96	25.00	111.07
**16**	3.31	0.40	5.04	0.63	0.66	0.27	0.25	21.37	94.43	36.78	56.66	39.76	61.63

Table S5. Pearson pairwise correlations of macronutrients, micronutrients and silver in tomato stem.

Variable 1	Variable 2	N	r	IC de 95% para *p*	*p*
Ca (%)	Na (%)	16	0.005	(-0.492, 0.500)	0.985
Mg (%)	Na (%)	16	-0.190	(-0.627, 0.337)	0.480
S (%)	Na (%)	16	0.405	(-0.113, 0.750)	0.119
Cu (mg Kg^{-1})	Na (%)	16	0.050	(-0.457, 0.532)	0.855
Fe (mg Kg^{-1})	Na (%)	16	0.396	(-0.123, 0.746)	0.128
Mn (mg Kg^{-1})	Na (%)	16	-0.021	(-0.511, 0.480)	0.939
Zn (mg Kg^{-1})	Na (%)	16	0.274	(-0.256, 0.678)	0.304
B (mg Kg^{-1})	Na (%)	16	0.128	(-0.393, 0.586)	0.637
Ag (mg Kg^{-1})	Na (%)	16	-0.008	(-0.502, 0.490)	0.976
N (%)	Na (%)	16	-0.357	(-0.725, 0.168)	0.175
P (%)	Na (%)	16	0.489	(-0.008, 0.793)	0.054
K (%)	Na (%)	16	0.422	(-0.093, 0.759)	0.103
Mg (%)	Ca (%)	16	0.269	(-0.262, 0.675)	0.314
S (%)	Ca (%)	16	-0.073	(-0.549, 0.438)	0.787
Cu (mg Kg^{-1})	Ca (%)	16	0.014	(-0.485, 0.506)	0.959
Fe (mg Kg^{-1})	Ca (%)	16	0.282	(-0.249, 0.682)	0.291
Mn (mg Kg^{-1})	Ca (%)	16	0.170	(-0.356, 0.614)	0.530
Zn (mg Kg^{-1})	Ca (%)	16	-0.278	(-0.680, 0.252)	0.297
B (mg Kg^{-1})	Ca (%)	16	-0.277	(-0.679, 0.254)	0.299
**Ag (mg Kg^{-1})**	**Ca (%)**	**16**	**-0.523**	**(-0.809, -0.037)**	**0.038 ***
N (%)	Ca (%)	16	0.082	(-0.431, 0.555)	0.763
P (%)	Ca (%)	16	0.220	(-0.309, 0.645)	0.412
K (%)	Ca (%)	16	0.377	(-0.146, 0.735)	0.150
S (%)	Mg (%)	16	-0.517	(-0.806, -0.028)	0.040
Cu (mg Kg^{-1})	Mg (%)	16	0.112	(-0.406, 0.576)	0.680
Fe (mg Kg^{-1})	Mg (%)	16	0.512	(0.022, 0.804)	0.042
Mn (mg Kg^{-1})	Mg (%)	16	0.076	(-0.436, 0.551)	0.778

Zn (mg Kg^{-1})	Mg (%)	16	-0.253	(-0.665, 0.277)	0.344
B (mg Kg^{-1})	Mg (%)	16	-0.137	(-0.592, 0.385)	0.614
Ag (mg Kg^{-1})	Mg (%)	16	-0.322	(-0.705, 0.207)	0.225
N (%)	Mg (%)	16	0.132	(-0.389, 0.589)	0.625
P (%)	Mg (%)	16	0.306	(-0.224, 0.696)	0.249
K (%)	Mg (%)	16	-0.335	(-0.712, 0.193)	0.205
Cu (mg Kg^{-1})	S (%)	16	-0.119	(-0.580, 0.400)	0.661
Fe (mg Kg^{-1})	S (%)	16	-0.123	(-0.583, 0.397)	0.650
Mn (mg Kg^{-1})	S (%)	16	-0.508	(-0.802, -0.017)	0.044
Zn (mg Kg^{-1})	S (%)	16	0.017	(-0.483, 0.508)	0.951
B (mg Kg^{-1})	S (%)	16	0.084	(-0.429, 0.557)	0.757
Ag (mg Kg^{-1})	S (%)	16	0.214	(-0.315, 0.642)	0.425
N (%)	S (%)	16	-0.004	(-0.499, 0.492)	0.987
P (%)	S (%)	16	0.260	(-0.270, 0.670)	0.330
K (%)	S (%)	16	0.350	(-0.177, 0.720)	0.184
Fe (mg Kg^{-1})	Cu (mg Kg^{-1})	16	0.014	(-0.485, 0.506)	0.959
Mn (mg Kg^{-1})	Cu (mg Kg^{-1})	16	-0.102	(-0.569, 0.415)	0.708
Zn (mg/Kg)	Cu (mg Kg^{-1})	16	-0.295	(-0.690, 0.235)	0.267
B (mg Kg^{-1})	Cu (mg Kg^{-1})	16	-0.304	(-0.695, 0.226)	0.253
Ag (mg Kg^{-1})	Cu (mg Kg^{-1})	16	-0.075	(-0.550, 0.437)	0.783
N (%)	Cu (mg Kg^{-1})	16	-0.180	(-0.620, 0.347)	0.505
P (%)	Cu (mg Kg^{-1})	16	0.121	(-0.398, 0.582)	0.654
K (%)	Cu (mg Kg^{-1})	16	-0.271	(-0.676, 0.259)	0.310
Mn (mg Kg^{-1})	Fe (mg Kg^{-1})	16	0.232	(-0.298, 0.653)	0.386
Zn (mg Kg^{-1})	Fe (mg Kg^{-1})	16	0.227	(-0.303, 0.649)	0.399
B (mg Kg^{-1})	Fe (mg Kg^{-1})	16	-0.155	(-0.605, 0.369)	0.566
Ag (mg Kg^{-1})	Fe (mg Kg^{-1})	16	-0.143	(-0.597, 0.379)	0.596
N (%)	Fe (mg Kg^{-1})	16	-0.174	(-0.616, 0.352)	0.520
P (%)	Fe (mg Kg^{-1})	16	0.492	(-0.005, 0.794)	0.053
K (%)	Fe (mg Kg^{-1})	16	0.115	(-0.404, 0.578)	0.672

Zn (mg Kg^{-1})	Mn (mg Kg^{-1})	16	0.505	(0.012, 0.800)	0.046
B (mg Kg^{-1})	Mn (mg Kg^{-1})	16	-0.031	(-0.519, 0.472)	0.910
Ag (mg Kg^{-1})	Mn (mg Kg^{-1})	16	-0.025	(-0.514, 0.477)	0.928
N (%)	Mn (mg Kg^{-1})	16	-0.043	(-0.527, 0.463)	0.875
P (%)	Mn (mg Kg^{-1})	16	0.200	(-0.328, 0.633)	0.458
K (%)	Mn (mg Kg^{-1})	16	0.143	(-0.379, 0.597)	0.597
B (mg Kg^{-1})	Zn (mg Kg^{-1})	16	0.240	(-0.291, 0.657)	0.372
**Ag (mg Kg^{-1})**	**Zn (mg Kg^{-1})**	**16**	**0.601**	**(0.150, 0.845)**	**0.014 ***
N (%)	Zn (mg Kg^{-1})	16	0.153	(-0.371, 0.603)	0.572
P (%)	Zn (mg Kg^{-1})	16	-0.047	(-0.531, 0.459)	0.862
K (%)	Zn (mg Kg^{-1})	16	0.045	(-0.461, 0.529)	0.868
Ag (mg/Kg)	B (mg Kg^{-1})	16	0.254	(-0.276, 0.666)	0.342
N (%)	B (mg Kg^{-1})	16	-0.036	(-0.523, 0.468)	0.893
P (%)	B (mg Kg^{-1})	16	-0.219	(-0.644, 0.311)	0.416
K (%)	B (mg Kg^{-1})	16	0.110	(-0.408, 0.575)	0.684
N (%)	Ag (mg Kg^{-1})	16	-0.139	(-0.594, 0.383)	0.608
P (%)	Ag (mg Kg^{-1})	16	-0.325	(-0.707, 0.203)	0.219
K (%)	Ag (mg Kg^{-1})	16	-0.470	(-0.783, 0.034)	0.066
P (%)	N (%)	16	-0.163	(-0.610, 0.362)	0.546
K (%)	N (%)	16	0.170	(-0.355, 0.614)	0.528
K (%)	P (%)	16	0.356	(-0.169, 0.724)	0.176

Pearson correlation coefficients (r). * Significant values ($p \leq 0.05$).
